# Supplementary material for: Effects of human disturbances on wildlife behaviour and consequences for predator-prey overlap in Southeast Asia
Source: Nat Commun. 2024 Feb 19;15:1521. doi: 10.1038/s41467-024-45905-9 (PMC10876642; doi:10.1038/s41467-024-45905-9)
Supplement: Supplementary file 1 — Supplementary Information [file 41467_2024_45905_MOESM1_ESM.pdf]

Effects of human disturbances on wildlife behaviour and consequences for predator-prey overlap in Southeast Asia

Authors: Samuel Xin Tham Lee[1], Zachary Amir[1], Jonathan H. Moore[2,3], Kaitlyn M. Gaynor[4], and Matthew Scott Luskin[5,6]*

Affiliations:

[1] School of the Environment, University of Queensland, Brisbane, Australia.

[2] School of Environmental Science and Engineering, Southern University of Science and Technology, Shenzhen, China.

[3] School of Environmental Sciences, University of East Anglia, Norwich, United Kingdom.

[4] Departments of Zoology and Botany, University of British Columbia, British Columbia, Canada.

[5] Institute of the Environment and Sustainability, University of California, Los Angeles, United States.

[6] Centre for Biodiversity and Conservation Science, University of Queensland, Brisbane, Australia.

Supplementary Information

Supplementary Figures

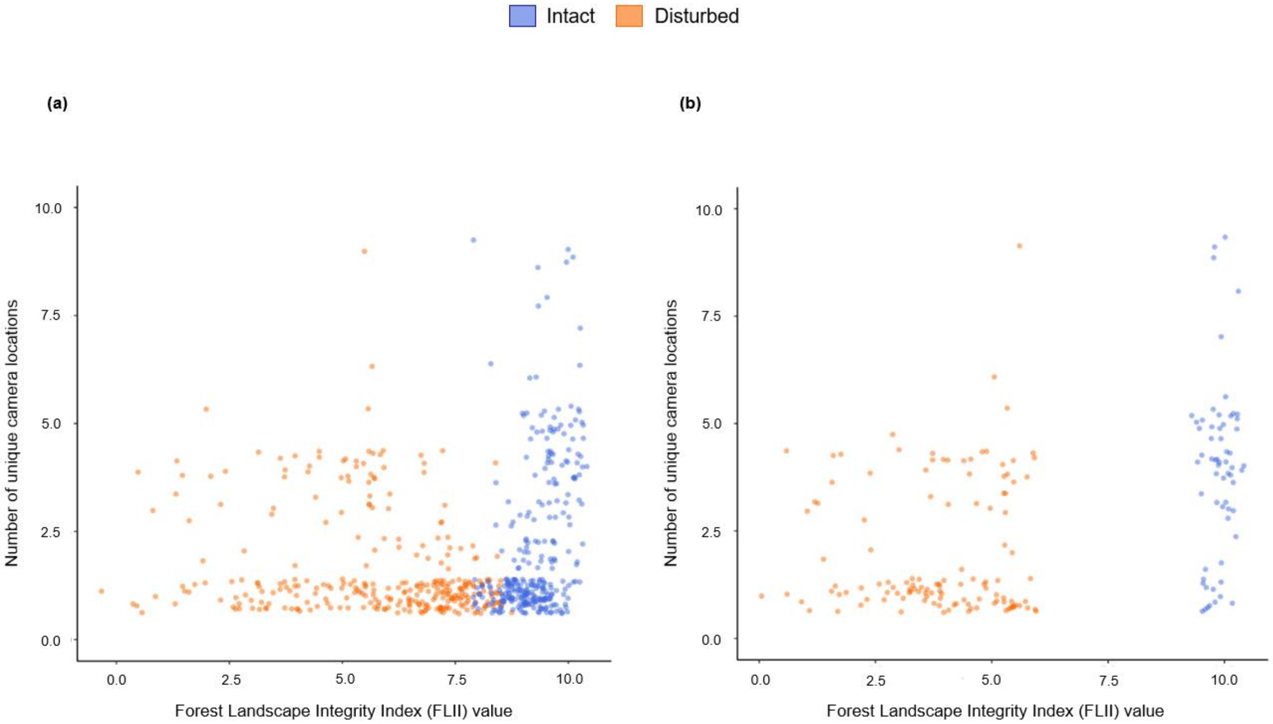

Supplementary Figure 1. Forest Landscape Integrity Index scores for 1218 cameras were included in this study. Cameras were grouped into roughly equal sample sizes of intact habitats (blue) or disturbed habitats (orange) based on the median ($FLII_{median}$ = 8.77) (**a**) or by the first (Q1 = 5.61) and third (Q3 = 9.73) quartiles of the forest integrity distribution (**b**). A jitter was applied to show overlapping points. Not shown in the plots are 46 unique cameras with forest integrity of 0 that were included in all analyses.

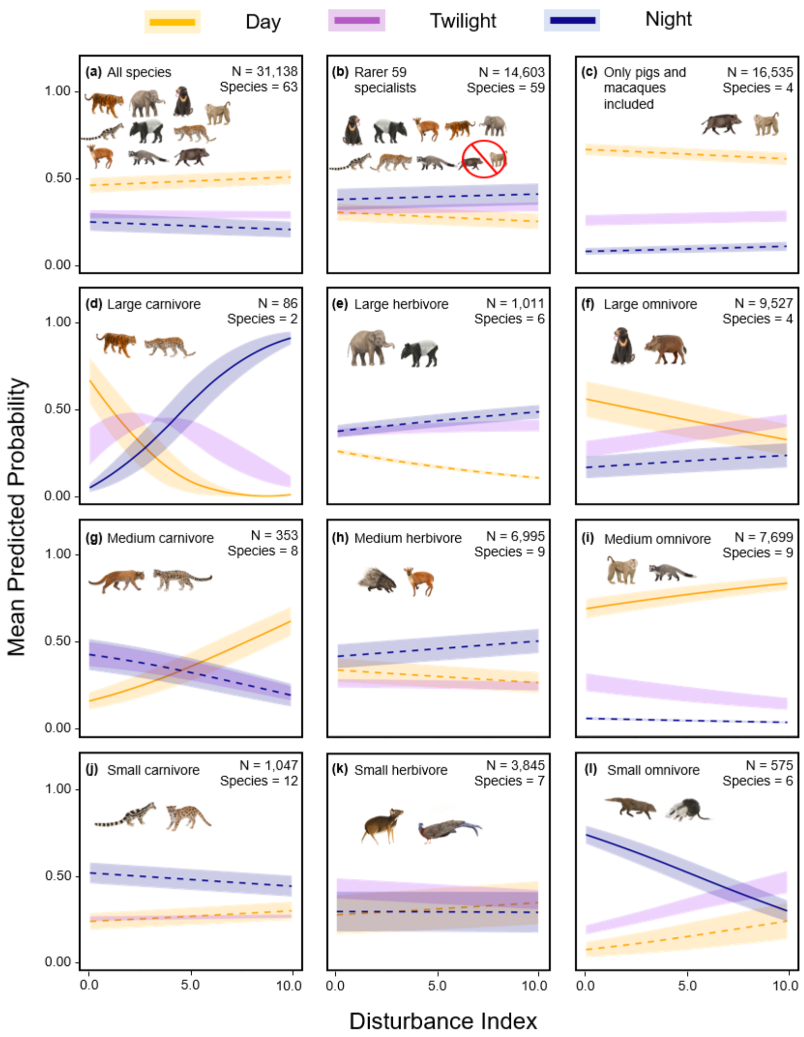

Supplementary Figure 2. Influence of humans and forest disturbance on the diel activity of tropical vertebrate communities and guilds, excluding species-level random effect and thereby assessing combined effects of within-species behavioural changes and species replacements within guilds. Interpretation is the same as for Fig. 4 in the main text.

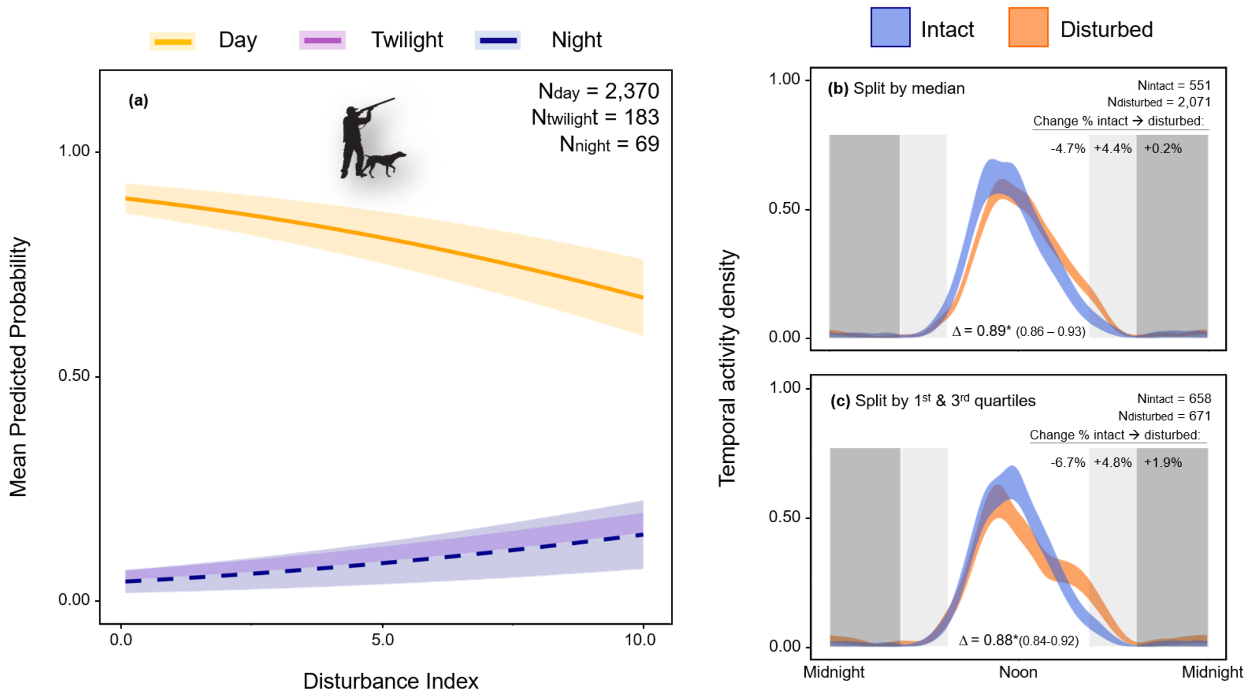

Supplementary Figure 3. Human activity in Southeast Asian tropical forests, estimated using multinomial regressions (a) and kernel density (b-c). The disturbance index is the inverse of the forest landscape integrity index (FLII; Grantham *et al.* 2020). Statistics for N_{day}, $N_{twilight}$, and N_{night} are the independent detections per time period. Trend lines correspond to the change in mean predicted probabilities of diel activity occurring during the day, twilight and night (orange, purple and blue lines, respectively). Interpretations match the corresponding figure types in the main text. In panel **(b)**, the ribbons show detections at 50% least (blue) and most disturbed forest cameras (orange; median FLII of 8.77) while panel **(c)** shows cameras in the least disturbed 25% of cameras (blue, FLII > 9.73) versus the most disturbed (orange, FLII < 5.61, 1st Quartile).

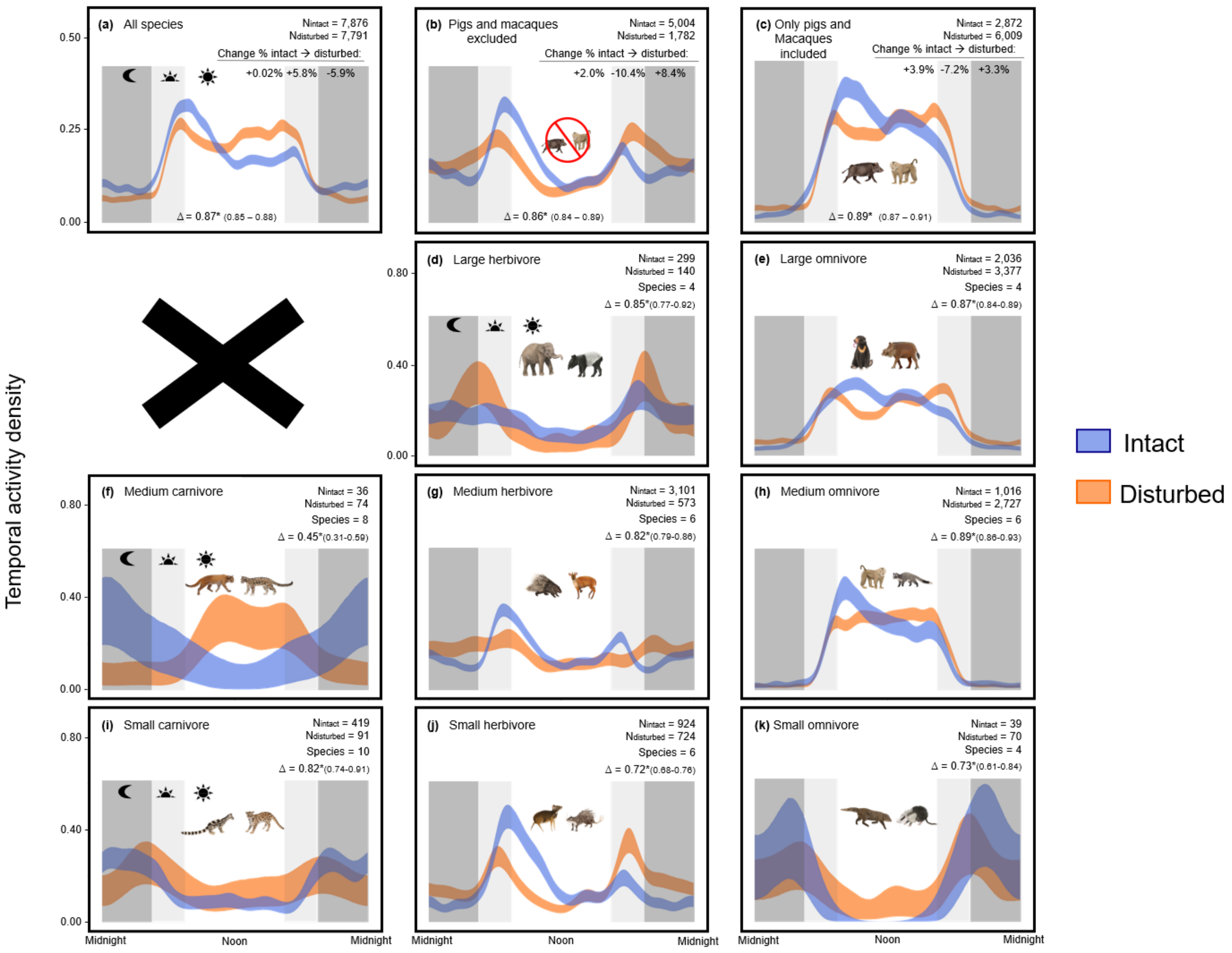

Temporal activity density
(a) All species
Nintact = 7,876
Ndisturbed = 7,791
Change % intact → disturbed:
+0.02% +5.8% -5.9%
Δ = 0.87* (0.85 – 0.88)
(b) Pigs and macaques excluded
Nintact = 5,004
Ndisturbed = 1,782
Change % intact → disturbed:
+2.0% -10.4% +8.4%
Δ = 0.86* (0.84 – 0.89)
(c) Only pigs and Macaques included
Nintact = 2,872
Ndisturbed = 6,009
Change % intact → disturbed:
+3.9% -7.2% +3.3%
Δ = 0.89* (0.87 – 0.91)
(d) Large herbivore
Nintact = 299
Ndisturbed = 140
Species = 4
Δ = 0.85*(0.77-0.92)
(e) Large omnivore
Nintact = 2,036
Ndisturbed = 3,377
Species = 4
Δ = 0.87*(0.84-0.89)
(f) Medium carnivore
Nintact = 36
Ndisturbed = 74
Species = 8
Δ = 0.45*(0.31-0.59)
(g) Medium herbivore
Nintact = 3,101
Ndisturbed = 573
Species = 6
Δ = 0.82*(0.79-0.86)
(h) Medium omnivore
Nintact = 1,016
Ndisturbed = 2,727
Species = 6
Δ = 0.89*(0.86-0.93)
(i) Small carnivore
Nintact = 419
Ndisturbed = 91
Species = 10
Δ = 0.82*(0.74-0.91)
(j) Small herbivore
Nintact = 924
Ndisturbed = 724
Species = 6
Δ = 0.72*(0.68-0.76)
(k) Small omnivore
Nintact = 39
Ndisturbed = 70
Species = 4
Δ = 0.73*(0.61-0.84)
0.00
0.25
0.50
0.40
0.80
Midnight
Noon
Midnight
Intact
Disturbed

Supplementary Figure 4. Wildlife and human activity in 25% most intact (blue) and 25% most disturbed forests (orange). To assess the sensitivity of our results to the splitting cameras into low vs high disturbance using the median forest integrity, we reanalysed activity patterns comparing by 1st and 3rd quartiles of forest disturbance (i.e., very intact vs very disturbed). This more extreme split exhibited qualitatively similar results but more pronounced changes. For example, when comparing 1st and 3rd quartiles, all species showed a +5.8% increase in crepuscular detections in the most disturbed forests, compared to +2.0% when splitting at the median forest disturbance. When excluding pigs and macaques, the new quartiles split showed +8.4% nocturnal detections for the other 59 species in the most disturbed forest compared to +6.9% when splitting by median forest disturbance. Similar to the community-level sensitivity analyses, the extreme split led to guild-level activity patterns that also remained qualitatively similar but with more pronounced activity peaks and shifts. For example, medium carnivores increased their diurnal detections by +51% within the most disturbed forests compared to +25.8% when splitting by median forest disturbance. The exception was large and small herbivores that displayed similar trends with both splits, such as +2.3 % and +6.8% increase in crepuscular detections respectively within the most disturbed forests compared to -1.6% and -0.1% decrease respectively when splitting by median forest disturbance. Interpretations follow kernel density analyses and Figure 3 from the main text. Large carnivores were excluded from this analysis (× symbol) due to insufficient detections. The percentage change in detections per guild in diurnal, crepuscular and nocturnal periods is shown in Supplementary Table 5.

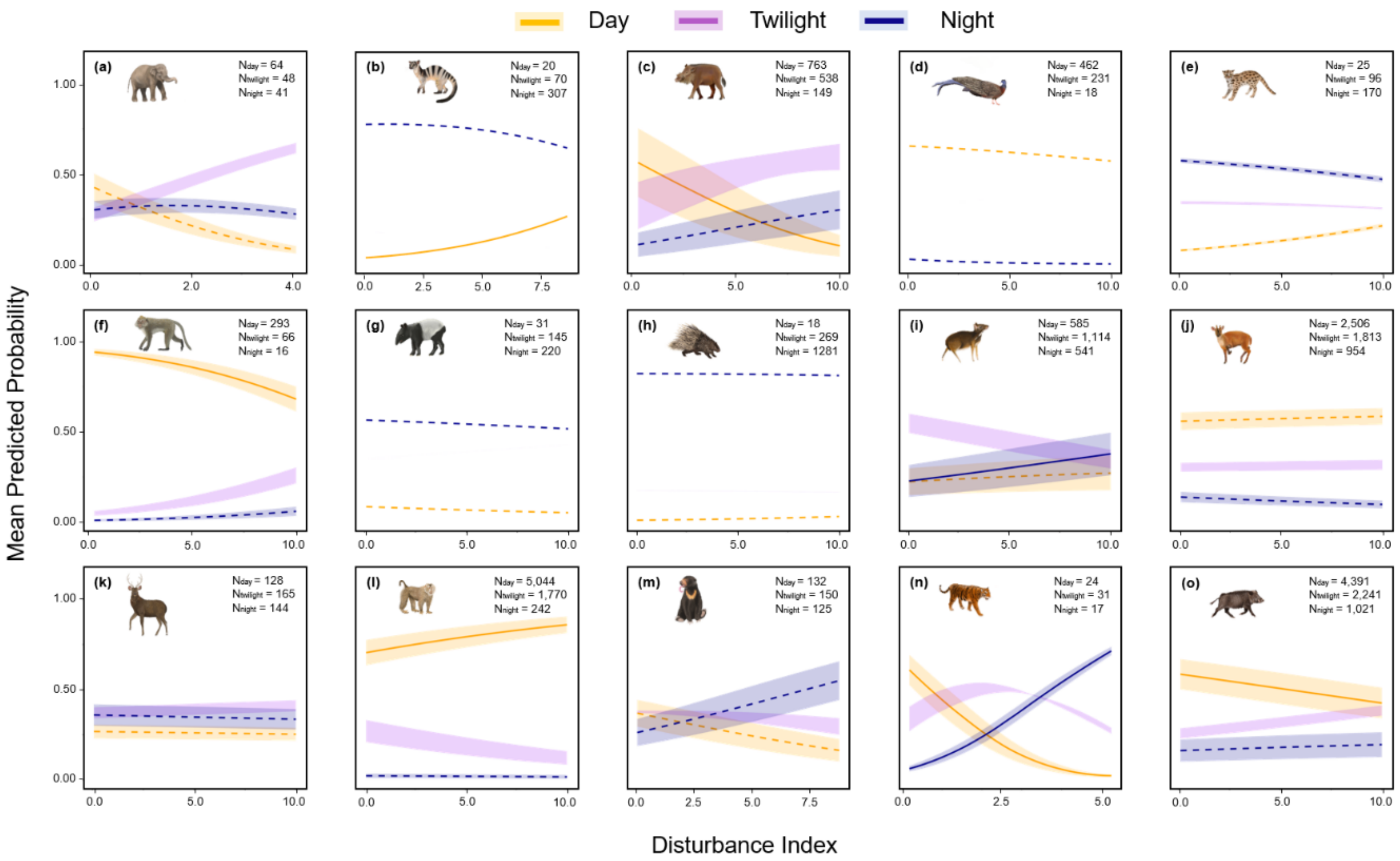

Supplementary Figure 5. Influence of humans and forest degradation on the diel activity of 15 vertebrate species in Southeast Asia. Panels show results for species with ≥15 detections in all three time periods, including **(a)** Asian elephant (*Elephas maximus*), **(b)** banded civet (*Hemigalus derbyanus*), **(c)** bearded pig (*Sus* barbatus), **(d)** great argus (*Argusianus argus*), **(e)** leopard cat (*Prionailurus spp.*), **(f)** long-tailed macaque (*Macaca fascicularis*), **(g)** Malay tapir (*Tapirus indicus*), **(h)** Malayan porcupine (*Hystrix brachyurus*), **(i)** mouse deer (*Tragulus spp.*), **(j)** red muntjac (*Muntiacus muntjak*), **(k)** sambar deer (*Rusa unicolor*), **(l)** southern pig-tailed macaque (*Macaca nemestrina*), **(m)** sun bear (*Helarctos malayanus*), **(n)** tiger (*Panthera tigris*), and **(o)** wild boar (*Sus scrofa*). Interpretations follow Fig. 4 in the main text.

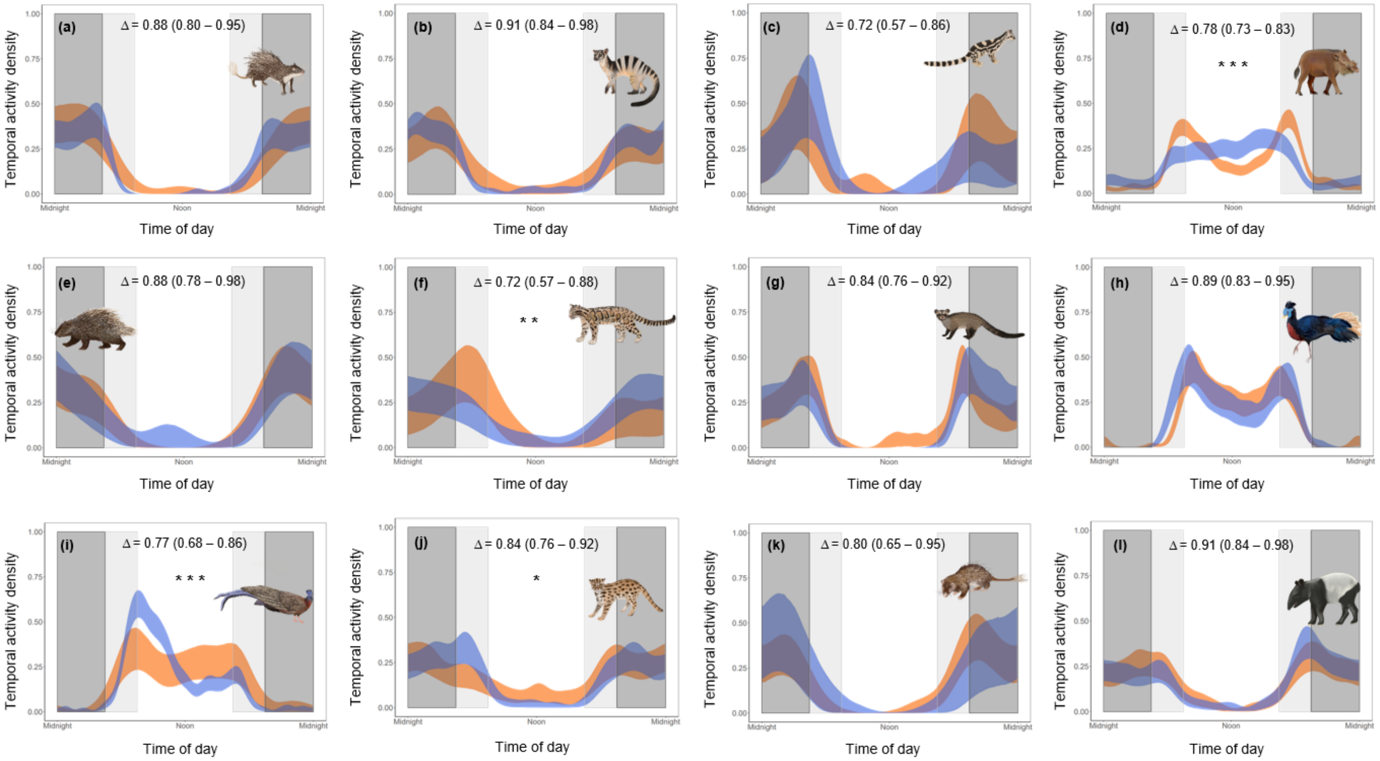

(a) Δ = 0.88 (0.80 – 0.95)
(b) Δ = 0.91 (0.84 – 0.98)
(c) Δ = 0.72 (0.57 – 0.86)
(d) Δ = 0.78 (0.73 – 0.83) * * *
(e) Δ = 0.88 (0.78 – 0.98)
(f) Δ = 0.72 (0.57 – 0.88) * *
(g) Δ = 0.84 (0.76 – 0.92)
(h) Δ = 0.89 (0.83 – 0.95)
(i) Δ = 0.77 (0.68 – 0.86) * * *
(j) Δ = 0.84 (0.76 – 0.92) *
(k) Δ = 0.80 (0.65 – 0.95)
(l) Δ = 0.91 (0.84 – 0.98)
Temporal activity density
Time of day
Midnight
Noon
Midnight
0.00
0.25
0.50
0.75
1.00

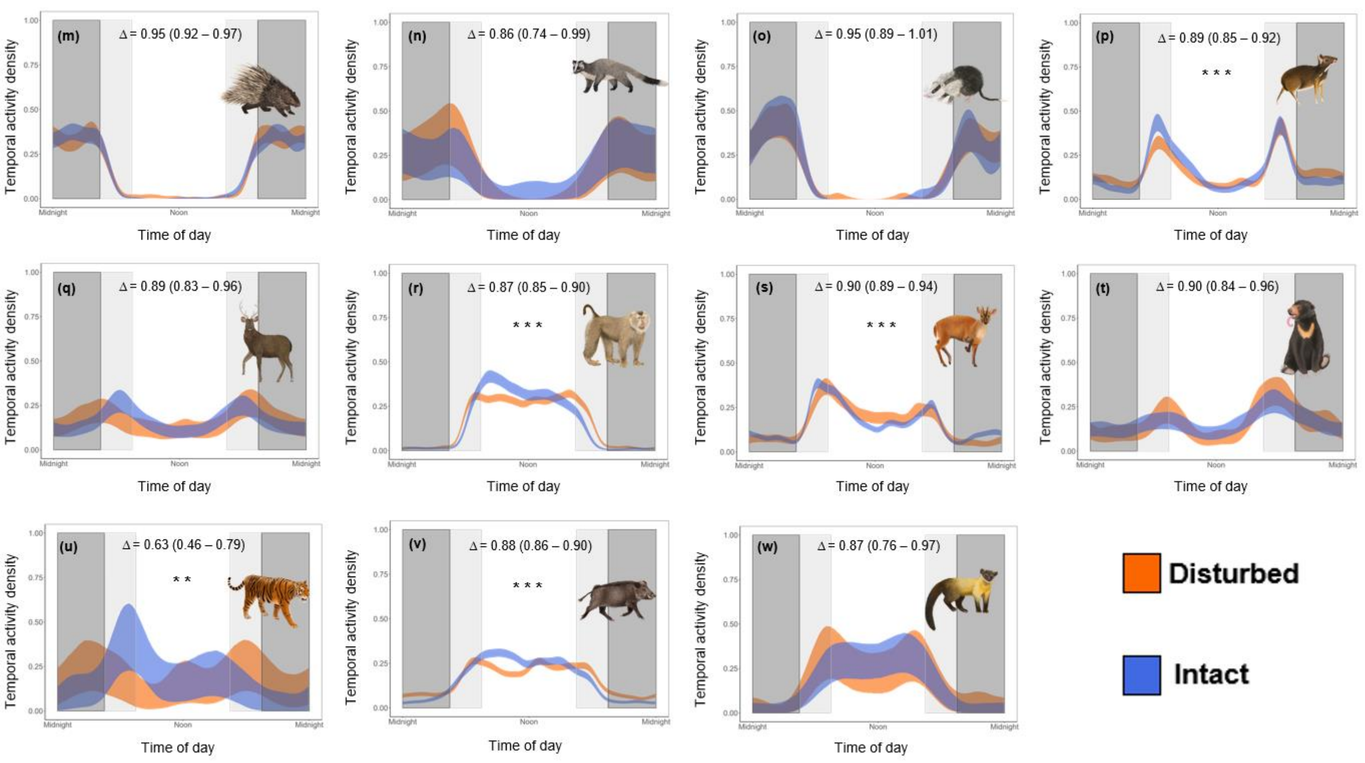

Supplementary Figure 6. Activity patterns of 23 wildlife species in disturbed and intact forests of Southeast Asia. The 23 species includes the Asiatic brush-tailed porcupine **(a)**, banded civet **(b)**, banded linsang **(c)**, bearded pig **(d)**, Bornean porcupine **(e)**, clouded leopard **(f)**, common palm civet **(g)**, crested fireback pheasant **(h)**, great argus **(i)**, leopard cat **(j)**, long-tailed porcupine **(k)**, Malay tapir **(l)**, Malayan porcupine **(m)**, masked palm civet **(n)**, moon rat **(o)**, mouse deer **(p)**, sambar deer **(q)**, southern pig-tailed macaque **(r)**, southern red muntjac **(s)**, sun bear **(t)**, tiger **(u)**, wild boar **(v)**, and yellow-throated marten **(w)**. Ribbons show 95% confidence intervals from bootstrapped kernel activity densities functions. Blue ribbon shows intact forest (FLII > 8.77) and orange shows disturbed sites (FLII ≤ 8.77). "Δ" denotes the overlap or shared area found between the two activity distributions. The "*" denotes the p-value for each comparison where "*" indicates $0.01 < p \leq 0.05$, "**" indicates $0.001 < p \leq 0.01$, "***" indicates $p \leq 0.001$ and " " indicates non-significance ($p \geq 0.05$). Background shading denotes diurnal hours (white), crepuscular hours (light grey) and nocturnal hours (dark grey). For exact P-values, please refer to Supplementary Table 19.

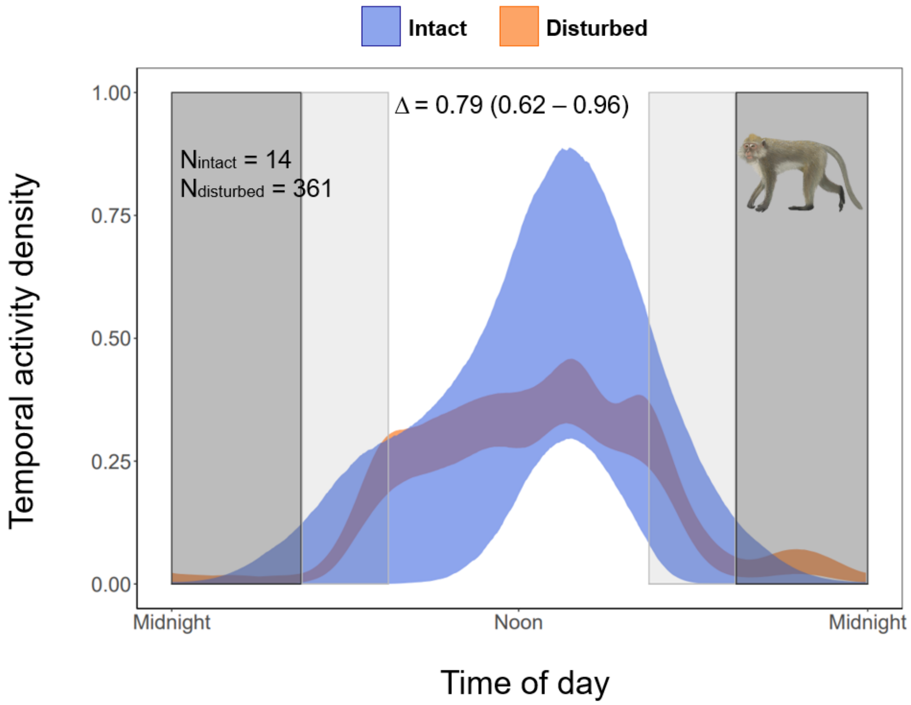

Supplementary Figure 7. Activity pattern the common long-tailed macaque between intact and disturbed forests. Ribbons show 95% confidence intervals from bootstrapped kernel activity densities functions. Blue ribbon shows intact forest (FLII > 8.77) and orange shows disturbed sites (FLII ≤ 8.77). "Δ" denotes the overlap or shared area found between the two activity distributions. The shift was non-significant ($P = 0.67$). Background shading denotes diurnal hours (white), crepuscular hours (light grey) and nocturnal hours (dark grey).

Supplementary Tables

Supplementary Table 1. **Study site description and effort for new camera trapping**. Trap nights were estimated between each collected camera's first and last photos. MCP refers to the minimum convex polygon around the camara traps. To account for variation in deployment scale and spacing, we resampled all data by grouping cameras into 1-km apothem hexagonal units (3.45 km^2 cells). Therefore, the rows in the capture histories all represent the same sampling area, and because some cells had more than one camera, we included trapping effort per cell per sampling window as a covariate of detection.

Survey	Annual rainfall	Cameras collected	Effort (trap nights)	Duration	Elevation (Mean ± SD)	Elevation range	MCP	Camera spacing
**THAILAND**								
**Khao Chong / Khao Ban Tat 2018**	2014.28	76	3957	2018-02-01 – 2018-04-30	524.59 ± 270.92	103 – 1234	59.01	467.95
**Khao Yai 2019**	1119.49	61	3553	2019-07-01 – 2019-09-25	769.64 ± 38.56	582 – 816	22.54	464.42
**SUMATRA**								
**Gunung Leuser 2014**	2828.00	69	3401	2013-12-18 – 2014-05-22	316.03 ± 250.38	25 – 888	516.15	1275.27
**Kerinci Seblat 2014**	2406.94	98	5356	2014-02-10 – 2014-10-04	594.03 ± 194.46	252 – 1154	813.69	1169.04
**Bukit Barisan Selatan 2014**	2987.80	79	5750	2014-06-15 – 2014-09-20	369.75 ± 184.97	116 – 935	473.58	1139.96
**MALAYSIAN BORNEO**								
**Danum Valley 2019 (Sabah)**	2182.68	22	1292	2019-05-24 – 2019-09-26	256.73 ± 102.02	184 – 567	8.31	520.76
**Danum Valley 2018 (Sabah)**	2182.85	27	1849	2018-07-12 – 2018-10-30	249.63 ± 53.23	175 – 381	15.95	614.15

**Lambir Hills 2017 (Sarawak**	3078.82	67	2406	2017-05-23 – 2017-07-07	164.80 ± 65.31	60.31 – 421.44	22.06	459.95
**PENINSULAR MALAYSIA**								
**Pasoh 2013**	2081.40	58	1399	2013-05-29 – 2014-02-12	297.09 ± 160.10	98 – 674	133.53	1316.26
**Pasoh 2014**	2079.16	57	1314	2014-05-13 – 2014-08-01	303.14 ± 160.04	98 – 674	134.62	1321.48
**Pasoh 2015**	2079.78	59	1670	2015-05-07 – 2015-09-04	301.15 ± 158.15	98 – 674	134.62	1317.78
**Pasoh 2016**	2086.38	42	1305	2017-05-17 – 2017-08-29	308.98 ± 156.05	103 – 674	122.63	1416.43
**Ulu Muda 2015a**	2057.03	76	4242	2014-11-01 – 2015-01-30	278.58 ± 128.68	117 – 628	68.98	938.65
**Ulu Muda 2015b**	2063.01	112	4446	2015-01-31 – 2015-05-01	295.77 ± 139.83	117-843	113.61	731.56
**Ulu Muda 2015c**	2080.90	52	3582	2015-05-02 – 2015-07-31	325.38 ± 166.15	141-843	115.53	1227.86
**Ulu Muda 2015d**	2078.17	48	2862	2015-08-01 – 2015-10-30	328.92 ± 165.27	123-843	104.01	1237.85
**Ulu Muda 2016a**	2065.89	73	2220	2015-10-31 – 2016-01-29	313.62 ± 145.27	117-748	103.17	794.92
**Ulu Muda 2016b**	2054.55	60	2899	2016-01-30 – 2016-04-29	285.45 ± 135.12	117-628	66.96	958.39
**Ulu Muda 2016c**	2060.54	46	2746	2016-04-30 – 2016-07-22	301.30 ± 138.91	117-628	65.72	974.47
**SINGAPORE**								
**Singapore 2019**	2283.97	36	2359	2018-12-26 – 2019-03-17	41.44 ± 22.18	0 – 83	162.35	261.70

Supplementary Table 2. Species selected for the community and guild-level temporal shift analyses. Each species' total number of detections was pooled across 10 forested landscapes in Southeast Asia. The 'IUCN' column refers to the IUCN Conservation Status of the species (*i.e.*, 'LC' = Least Concern, 'NT' = Near Threatened, 'VU' = Vulnerable, 'EN' = Endangered, 'CR' = Critically Endangered, 'DD' = Data Deficient). The 'Landscape detected' column refers to the number of landscapes that species was detected. Lastly, the 'Trophic guild' column refers to the trophic level occupied by a species based on diet and body mass (Refer to section 2.3 of methods for assigning guilds).

Common name	Scientific name	IUCN	Landscape detected	Total detections	Feeding guild	Body mass (kg)	Trophic guild
Leopard	*Panthera pardus*	VU	2	14	Carnivore	52	Large carnivore
Tiger	*Panthera tigris*	EN	3	72	Carnivore	163	Large carnivore
Gaur	*Bos gaurus*	VU	1	8	Herbivore	665	Large herbivore
Chinese serow	*Capricornis milneedwardsii*	NT	1	6	Herbivore	150	Large herbivore
Mainland serow	*Capricornis sumatraensis*	VU	2	11	Herbivore	80	Large herbivore
Asian elephant	*Elephas maximus*	EN	5	153	Herbivore	3320	Large herbivore
Sambar deer	*Rusa unicolor*	VU	8	437	Herbivore	170	Large herbivore
Malay tapir	*Tapirus indicus*	EN	5	396	Herbivore	308	Large herbivore
Sun bear	*Helarctos malayanus*	VU	8	407	Omnivore	57	Large omnivore
Bearded pig	*Sus barbatus*	VU	4	1450	Omnivore	135	Large omnivore
Wild boar	*Sus scrofa*	VU	8	7654	Omnivore	85	Large omnivore
Asiatic black bear	*Ursus thibetanus*	VU	3	16	Omnivore	100	Large omnivore
Asiatic golden cat	*Catopuma temminckii*	NT	4	31	Carnivore	7.7	Medium carnivore
Dhole	*Cuon alpinus*	EN	2	30	Carnivore	16	Medium carnivore
Clouded leopard	*Neofelis spp.*	VU	7	113	Carnivore	15	Medium carnivore
Clouded monitor	*Varanus nebulosus*	NT	1	38	Carnivore	4.3	Medium carnivore

Common water monitor	*Varanus salvator*	LC	4	32	Carnivore	20	Medium carnivore
Large spotted civet	*Viverra megaspila*	EN	2	4	Carnivore	9.1	Medium carnivore
Malay civet	*Viverra tangalunga*	LC	4	59	Carnivore	7.4	Medium carnivore
Large Indian civet	*Viverra zibetha*	LC	3	46	Carnivore	9.2	Medium carnivore
Malayan porcupine	*Hystrix brachyura*	LC	9	1568	Herbivore	8.0	Medium herbivore
Bornean porcupine	*Hystrix crassispinis*	LC	2	109	Herbivore	4.6	Medium herbivore
Sumatran mountain muntjac	*Muntiacus montanus*	DD	1	1	Herbivore	17.4	Medium herbivore
Southern red muntjac	*Muntiacus muntjak*	LC	9	5273	Herbivore	17.4	Medium herbivore
Yellow-handed mitered langur	*Presbytis melalophos*	EN	1	1	Herbivore	6.5	Medium herbivore
Red langur	*Presbytis rubicunda*	LC	4	21	Herbivore	6.4	Medium herbivore
White-thighed surili	*Presbytis siamensis*	NT	1	1	Herbivore	6.5	Medium herbivore
Siamang	*Symphalangus syndactylus*	EN	1	5	Herbivore	12	Medium herbivore
Dusky langur	*Trachypithecus obscurus*	NT	2	16	Herbivore	7.1	Medium herbivore
Binturong	*Arctictis binturong*	VU	9	54	Omnivore	13	Medium omnivore
Greater hog badger	*Arctonyx collaris*	VU	1	8	Omnivore	8.2	Medium omnivore
Sumatran hog badger	*Arctonyx hoevenii*	LC	2	11	Omnivore	8.2	Medium omnivore
Golden jackal	*Canis aureus*	LC	1	2	Omnivore	9.7	Medium omnivore
Stump-tailed macaque	*Macaca arctoides*	VU	1	76	Omnivore	9.4	Medium omnivore
Common long-tailed macaque	*Macaca fascicularis*	LC	7	375	Omnivore	4.6	Medium omnivore

Southern pig-tailed macaque	*Macaca nemestrina*	VU	9	7056	Omnivore	7.9	Medium omnivore
Sunda pangolin	*Manis javanica*	CR	3	41	Omnivore	4.9	Medium omnivore
Masked palm civet	*Paguma larvata*	LC	6	76	Omnivore	4.3	Medium omnivore
Banded civet	*Hemigalus derbyanus*	NT	8	397	Carnivore	1.3	Small carnivore
Crab-eating mongoose	*Herpestes urva*	LC	4	69	Carnivore	2.4	Small carnivore
Yellow-throated marten	*Martes flavigula*	LC	9	125	Carnivore	2.5	Small carnivore
Malay weasel	*Mustela nudipes*	LC	1	2	Carnivore	0.57	Small carnivore
Sunda stink badger	*Mydaus javanensis*	LC	1	36	Carnivore	2.5	Small carnivore
Changeable hawk eagle	*Nisaetus cirrhatus*	LC	1	1	Carnivore	1.5	Small carnivore
Marbled cat	*Pardofelis marmorata*	NT	7	42	Carnivore	2.8	Small carnivore
Leopard cat	*Prionailurus spp.*	LC	10	291	Carnivore	2.8	Small carnivore
Flat-headed cat	*Prionailurus planiceps*	EN	1	1	Carnivore	3.5	Small carnivore
Banded linsang	*Prionodon linsang*	LC	7	71	Carnivore	0.69	Small carnivore
House shrew	*Suncus murinus*	LC	1	7	Carnivore	0.043	Small carnivore
Small Indian civet	*Viverricula indica*	LC	1	5	Carnivore	3.0	Small carnivore
Great argus	*Argusianus argus*	VU	3	711	Herbivore	2.0	Small herbivore
Asiatic brush-tailed porcupine	*Atherurus macrourus*	LC	3	271	Herbivore	2.0	Small herbivore
Crested fireback pheasant	*Lophura spp.*	VU	5	522	Herbivore	1.8	Small herbivore
Black giant squirrel	*Ratufa bicolor*	NT	1	1	Herbivore	1.6	Small herbivore
Indomalayan bamboo rat	*Rhizomys sumatrensis*	LC	1	1	Herbivore	2.5	Small herbivore
Mouse deer	*Tragulus spp.*	LC	10	2240	Herbivore	3.6	Small herbivore
Long-tailed porcupine	*Trichys fasciculata*	LC	6	99	Herbivore	1.8	Small herbivore
Small-toothed palm civet	*Arctogalidia trivirgata*	LC	2	3	Omnivore	2.3	Small omnivore
Moon rat	*Echinosorex gymnura*	LC	7	281	Omnivore	1.0	Small omnivore
Short-tailed mongoose	*Herpestes brachyurus*	NT	5	23	Omnivore	1.4	Small omnivore
Javan mongoose	*Herpestes javanicus*	LC	2	2	Omnivore	0.77	Small omnivore
Bulwer's pheasant	*Lophura bulweri*	VU	1	6	Omnivore	1.2	Small omnivore
Common palm civet	*Paradoxurus hermaphroditus*	LC	7	260	Omnivore	3.2	Small omnivore

Supplementary Table 3. Species selected for species-level temporal shifts via the multinomial regression modelling approach. Each species' total number of detections was pooled across 10 forested landscapes in Southeast Asia. The “Day”, “Night”, and “Twilight” columns refer to the independent detections found within the “day” (0730 – 1630 hrs; 9 hours total), “night” (1930 – 0430 hrs; 9 hours total), and “twilight” (0430 – 0730 hrs or 1630 – 1930 hrs; 6 hours total) diel categories respectively. Only species with ≥15 detections in each diel category was included in this analysis.

Common name	Scientific name	Total detections	Day	Night	Twilight
Asian elephant	*Elephas maximus*	153	64	41	48
Banded civet	*Hemigalus derbyanus*	397	20	307	70
Bearded pig	*Sus barbatus*	1450	763	149	538
Common long-tailed macaque	*Macaca fascicularis*	375	293	16	66
Great argus	*Argusianus argus*	711	462	18	231
Leopard cat	*Prionailurus spp.*	291	25	170	96
Malay tapir	*Tapirus indicus*	396	31	220	145
Malayan porcupine	*Hystrix brachyura*	1568	18	1281	269
Mouse deer	*Tragulus spp.*	2240	585	541	1114
Sambar deer	*Rusa unicolor*	437	128	144	165
Southern pig-tailed macaque	*Macaca nemestrina*	7056	5044	242	1770
Southern red muntjac	*Muntiacus muntjak*	5273	2506	954	1813
Sun bear	*Helarctos malayanus*	407	132	125	150
Tiger	*Panthera tigris*	72	24	17	31
Wild boar	*Sus scrofa*	7654	4391	1021	2242

Supplementary Table 4. Species selected for species-level kernel density estimations as well as species pair overlap analyses. Each species' total number of detections was pooled across 10 forested landscapes in Southeast Asia. 'Intact detections' refer to total species detections within intact (FLII > 8.77) sites while 'Disturbed detections' refer to total species detections within disturbed (FLII ≤ 8.77) sites. 'Trophic guild' refers to the trophic level occupied by a species based on diet and body mass (Refer to section 2.3 of methods for assigning guilds).

Common name	Scientific name	Total detections	Intact detections	Disturbed detections	Feeding guild	Body mass (kg)	Trophic guild
Tiger	*Panthera tigris*	72	38	34	Carnivore	163	Large carnivore
Sambar deer	*Rusa unicolor*	437	273	164	Herbivore	170	Large herbivore
Malay tapir	*Tapirus indicus*	396	229	167	Herbivore	308	Large herbivore
Sun bear	*Helarctos malayanus*	407	231	176	Omnivore	57	Large omnivore
Wild boar	*Sus scrofa*	7654	3364	4290	Omnivore	84	Large omnivore
Bearded pig	*Sus barbatus*	1450	418	1032	Omnivore	135	Large omnivore
Clouded leopard	*Neofelis spp.*	113	73	40	Carnivore	15	Medium carnivore
Bornean porcupine	*Hystrix crassispinis*	109	45	64	Herbivore	4.6	Medium herbivore
Malayan porcupine	*Hystrix brachyura*	1568	867	701	Herbivore	8.0	Medium herbivore
Southern red muntjac	*Muntiacus muntjak*	5273	4264	1009	Herbivore	17	Medium herbivore
Masked palm civet	*Paguma larvata*	76	40	36	Omnivore	4.3	Medium omnivore
Pig-tailed macaque	*Macaca nemestrina*	7056	2201	4855	Omnivore	7.9	Medium omnivore
Banded linsang	*Prionodon linsang*	71	35	36	Carnivore	0.69	Small carnivore
Banded civet	*Hemigalus derbyanus*	397	317	80	Carnivore	1.3	Small carnivore
Yellow-throated marten	*Martes flavigula*	125	74	51	Carnivore	2.5	Small carnivore
Leopard cat	*Prionailurus spp.*	291	180	111	Carnivore	2.8	Small carnivore

Long-tailed porcupine	*Trichys fasciculata*	99	20	79	Herbivore	1.8	Small herbivore
Crested fireback pheasant	*Lophura spp.*	522	211	311	Herbivore	1.8	Small herbivore
Great argus	*Argusianus argus*	711	614	97	Herbivore	2.0	Small herbivore
Brush-tailed porcupine	*Atherurus macrourus*	271	196	75	Herbivore	2.0	Small herbivore
Mousedeer	*Tragulus sp.*	2240	1053	1187	Herbivore	3.6	Small herbivore
Moon rat	*Echinosorex gymnura*	281	123	158	Omnivore	1.0	Small omnivore
Common palm civet	*Paradoxurus hermaphroditus*	260	111	149	Omnivore	3.2	Small omnivore

Supplementary Table 5. Percentage change in diurnal, crepuscular and nocturnal detections from intact (FLII > 8.77) forests to disturbed (FLII ≤ 8.77) forests at the guild-level. Percentage change is calculated by the difference between percentage detections of a specific diel niche within disturbed forests versus intact forests, *i.e.*, (diurnal detections in disturbed habitats / total detections in disturbed habitats - diurnal detections in intact habitats / total detections in intact habitats) *100. The "Splitting method" column refers to how we define whether a camera site is "intact" or "disturbed" (refer to 2.2 of methods for clarification). The "large carnivore" guild is not included when splitting camera sites by the 1st and 3rd quantiles as there is insufficient sample size to perform the analysis (N < 20).

Splitting method	Guild	% Change in diurnal detections	% Change in crepuscular detections	% Change in nocturnal detections
Split by median FLII		-	-	-
	Large carnivore	-21.9%	+5.4%	+16.5%
	Large herbivore	-8.2%	-1.6%	+9.7%
	Large omnivore	-10.7%	+5.9%	+4.8%
	Medium carnivore	+25.8%	+0.4%	-26.2%
	Medium herbivore	-6.5%	-6.7%	+13.1%
	Medium omnivore	-7.1%	+8.2%	-1.1%
	Small carnivore	+5.3%	+0.7%	-5.9%
	Small herbivore	-7.3%	-0.1%	+7.3%
	Small omnivore	+6.1%	+3.9%	-10.0%
Split by Q1 and Q3 of FLII		-	-	-
	Large herbivore	-9.1%	+2.3%	+6.8%
	Large omnivore	-10.8%	+5.8%	+5.0%
	Medium carnivore	+51.0%	-8.9%	-42.1%
	Medium herbivore	-8.1%	-7.5%	+15.6%
	Medium omnivore	-1.4%	+2.8%	-1.3%

Small carnivore	+8.4%	+6.1%	-14.5%
Small herbivore	-23.2%	+6.8%	+16.3%
Small omnivore	+15.7%	+15.2%	-30.9%

Supplementary Table 6. Guild-level temporal outputs using kernel density estimation. 'AP_{Intact}' and '$AP_{Disturbed}$' signifies the activity peaks at intact (FLII > 8.77) and disturbed (FLII ≤ 8.77) sites respectively. P-values are estimated using the two-sided compareCkern test, however, no further statistical adjustments were made for each comparison (Note that the compareCkern function outputs "0" when P < 0.001, which we report here as "P < 0.001"). 'Δ (95% CI)' represents the estimated overlap coefficients and their associated 95% confidence interval. '1 - Δ' is the change in temporal overlap between intact and disturbed forests for a given trophic guild.

**Trophic guild**	**P-value**	**AP_{Intact}**	**$AP_{Disturbed}$**	**Δ (95% CI)**	**1-Δ**
Large carnivore	0.016	06:36 a.m.	17:26 p.m.	0.70 (0.55 – 0.84)	0.30
Large herbivore	0.023	18:39 p.m.	19:15 p.m.	0.90 (0.85 – 0.95)	0.10
Large omnivore	<0.001	08:54 a.m.	06:56 a.m.	0.88 (0.87 – 0.90)	0.12
Medium carnivore	<0.001	21:59 p.m.	18:14 p.m.	0.71 (0.63 – 0.80)	0.29
Medium herbivore	<0.001	06:36 a.m.	07:15 a.m.	0.85 (0.83 – 0.87)	0.15
Medium omnivore	<0.001	08:29 a.m.	15:59 p.m.	0.89 (0.87 – 0.91)	0.11
Small carnivore	0.11	00:56 a.m.	02:37 a.m.	0.92 (0.87 – 0.96)	0.08
Small herbivore	<0.001	06:53 a.m.	17:57 p.m.	0.86 (0.84 – 0.89)	0.14
Small omnivore	0.25	03:33 a.m.	03:45 a.m.	0.90 (0.85 – 0.95)	0.10

Supplementary Table 7. Multinomial logit model selection when all species were considered. "Diel category" refers to species detections found within each diel category (*i.e.*, day, twilight, and night), "disturbance" is the forest landscape integrity index (FLII), "body mass" is the mass of a species in kilograms, "body size" refers the body size categorisations given for each species [*i.e.*, large (>20 kg), medium (4-20 kg), and small (<4 kg)], and "feeding guild" is the typical diet of the species (*i.e.,* carnivore, herbivore, and omnivore). The Akaike Information Criterion (AIC) scores are ordered from the lowest (best models) to highest.

**Model with landscape random effect**	**AICc**
Diel category ~ disturbance * body size * feeding guild	58544.27
Diel category ~ disturbance * body mass * feeding guild	60424.23
Diel category ~ disturbance + body size + feeding guild	60485.16
Diel category ~ disturbance * feeding guild	60922.70
Diel category ~ feeding guild	61033.14
Diel category ~ disturbance + body mass + feeding guild	61034.59
Diel category ~ disturbance + feeding guild	61036.26
Diel category ~ disturbance * body size	62506.74
Diel category ~ disturbance + body size	63073.59
Diel category ~ body size	63074.39
Diel category ~ disturbance * body mass	63862.25
Diel category ~ disturbance + body mass	64023.00
Diel category ~ disturbance	64025.56
Diel category ~ body mass	64026.72
**Model with landscape and species random effect**	**AICc**
Diel category ~ disturbance * body size * feeding guild	53171.17
Diel category ~ disturbance * body size	53187.87
Diel category ~ disturbance * body mass * feeding guild	53215.89
Diel category ~ disturbance * body mass	53252.24
Diel category ~ disturbance * feeding guild	53301.70
Diel category ~ disturbance + body size	53319.77

Diel category ~ disturbance	53320.03
Diel category ~ disturbance + body mass	53320.42
Diel category ~ body mass	53324.00
Diel category ~ body size	53325.55
Diel category ~ disturbance + feeding guild	53325.95
Diel category ~ disturbance + body size + feeding guild	53326.67
Diel category ~ disturbance + body mass + feeding guild	53327.40
Diel category ~ feeding guild	53329.97

Supplementary Table 8. Model summary of the best multinomial logit model when all species were considered. The "twilight", "carnivore", and "small" were treated as the baseline or reference category in our analysis. The standard errors (SE) of coefficient estimates are in parentheses and P-values < 0.05 have an asterisk (*). Estimates of each model are obtained via the Restricted Maximum Likelihood (REML) method while p-values are estimated using the two-sided likelihood ratio test, however, no further statistical adjustments are made for each comparison.

Parameter from model with landscape random effect	Estimates (SE)	Z-value	P-value
Day (vs twilight) ~ (intercept)	-0.24 (0.15)	-1.58	0.12
Night (vs twilight) ~ (intercept)	1.05 (0.13)	7.99	$1.4*10^{-15}$*
Day (vs twilight) ~ disturbance	0.05 (0.05)	1.00	0.32
Night (vs twilight) ~ disturbance	-0.08 (0.04)	-1.86	0.064
Day (vs twilight) ~ body size(large)	0.48 (0.38)	1.27	0.21
Night (vs twilight) ~ body size(large)	-2.29 (0.51)	-4.48	$7.5*10^{-6}$*
Day (vs twilight) ~ body size(medium)	-0.88 (0.24)	-3.68	$2.2*10^{-4}$*
Night (vs twilight) ~ body size(medium)	-0.49 (0.19)	-2.57	0.010*
Day (vs twilight) ~ feeding guild(herbivore)	0.23 (0.12)	1.87	0.061
Night (vs twilight) ~ feeding guild(herbivore)	-1.70 (0.11)	-15.58	$9.8*10^{-55}$*
Day (vs twilight) ~ feeding guild(omnivore)	-1.67 (0.32)	-5.18	$2.3*10^{-7}$*
Night (vs twilight) ~ feeding guild(omnivore)	0.46 (0.18)	2.54	0.011*
Day (vs twilight) ~ disturbance*body size(large)	-0.31 (0.18)	-1.71	0.088
Night (vs twilight) ~ disturbance*body size(large)	0.37 (0.16)	2.27	0.023*
Day (vs twilight) ~ disturbance*body size(medium)	0.20 (0.07)	2.97	0.0030*
Night (vs twilight) ~ disturbance*body size(medium)	-0.05 (0.07)	-0.66	0.51
Day (vs twilight) ~ disturbance*feeding guild(herbivore)	-0.13 (0.05)	-2.61	0.010*
Night (vs twilight) ~ disturbance*feeding guild(herbivore)	0.13 (0.04)	2.99	0.0028*
Day (vs twilight) ~ disturbance*feeding guild(omnivore)	0.12 (0.09)	1.37	0.17
Night (vs twilight) ~ disturbance*feeding guild(omnivore)	-0.11 (0.07)	-1.68	0.093
Day (vs twilight) ~ body size(large)*feeding guild(herbivore)	-0.83 (0.40)	-2.08	0.037*

Night (vs twilight) ~ body size(large)*feeding guild(herbivore)	3.22 (0.52)	6.17	$6.8*10^{-10}$*
Day (vs twilight) ~ body size(medium)*feeding guild(herbivore)	0.99 (0.25)	3.98	$6.8*10^{-5}$*
Night (vs twilight) ~ body size(medium)*feeding guild(herbivore)	1.29 (0.21)	6.31	$2.8*10^{-10}$*
Day (vs twilight) ~ body size(large)*feeding guild(omnivore)	2.22 (0.49)	4.55	$5.5*10^{-6}$*
Night (vs twilight) ~ body size(large)*feeding guild(omnivore)	0.11 (0.54)	0.20	0.84
Day (vs twilight) ~ body size(medium)*feeding guild(omnivore)	3.84 (0.39)	9.89	$4.5*10^{-23}$*
Night (vs twilight) ~ body size(medium)*feeding guild(omnivore)	-2.28 (0.26)	-8.74	$2.4*10^{-18}$*
Day (vs twilight) ~ disturbance*body size(large)*feeding guild(herbivore)	0.30 (0.19)	1.60	0.11
Night (vs twilight) ~ disturbance*body size(large)*feeding guild(herbivore)	-0.44 (0.17)	-2.64	0.0083*
Day (vs twilight) ~ disturbance*body size(medium)*feeding guild(herbivore)	-0.07 (0.07)	-0.95	0.34
Night (vs twilight) ~ disturbance*body size(medium)*feeding guild(herbivore)	0.10 (0.08)	1.31	0.19
Day (vs twilight) ~ disturbance*body size(large)*feeding guild(omnivore)	0.09 (0.20)	0.45	0.66
Night (vs twilight) ~ disturbance*body size(large)*feeding guild(omnivore)	-0.18 (0.17)	-1.05	0.30
Day (vs twilight) ~ disturbance*body size(medium)*feeding guild(omnivore)	-0.32 (0.10)	-3.19	0.0014*
Night (vs twilight) ~ disturbance*body size(medium)*feeding guild(omnivore)	0.17 (0.09)	1.78	0.075
**Parameters from model with landscape and species random effect**	**Estimates (SE)**	**Z-value**	**P-value**
Day (vs twilight) ~ (intercept)	-0.47 (0.52)	-0.91	0.36
Night (vs twilight) ~ (intercept)	-0.08 (0.48)	-0.17	0.86
Day (vs twilight) ~ disturbance	0.10 (0.06)	1.76	0.079
Night (vs twilight) ~ disturbance	-0.01 (0.05)	-0.22	0.83
Day (vs twilight) ~ body size(large)	1.35 (1.29)	1.05	0.29
Night (vs twilight) ~ body size(large)	-1.89 (1.28)	-1.47	0.14
Day (vs twilight) ~ body size(medium)	-0.60 (0.80)	-0.75	0.45
Night (vs twilight) ~ body size(medium)	-0.15 (0.74)	-0.20	0.84
Day (vs twilight) ~ feeding guild(herbivore)	-0.50 (0.82)	-0.61	0.54
Night (vs twilight) ~ feeding guild(herbivore)	-0.65 (0.75)	-0.87	0.39
Day (vs twilight) ~ feeding guild(omnivore)	-0.53 (0.90)	-0.59	0.56
Night (vs twilight) ~ feeding guild(omnivore)	0.13 (0.84)	0.16	0.87
Day (vs twilight) ~ disturbance*body size(large)	-0.54 (0.22)	-2.44	0.015*

Night (vs twilight) ~ disturbance*body size(large)	0.43 (0.18)	2.37	0.018*
Day (vs twilight) ~ disturbance*body size(medium)	0.07 (0.10)	0.67	0.50
Night (vs twilight) ~ disturbance*body size(medium)	-0.05 (0.09)	-0.52	0.60
Day (vs twilight) ~ disturbance*feeding guild(herbivore)	-0.11 (0.06)	-1.85	0.064
Night (vs twilight) ~ disturbance*feeding guild(herbivore)	0.10 (0.05)	2.00	0.046*
Day (vs twilight) ~ disturbance*feeding guild(omnivore)	-0.03 (0.11)	-0.23	0.81
Night (vs twilight) ~ disturbance*feeding guild(omnivore)	-0.11 (0.07)	-1.57	0.12
Day (vs twilight) ~ body size(large)*feeding guild(herbivore)	-0.49 (1.58)	-0.31	0.76
Night (vs twilight) ~ body size(large)*feeding guild(herbivore)	2.59 (1.53)	1.69	0.090
Day (vs twilight) ~ body size(medium)*feeding guild(herbivore)	1.78 (1.18)	1.50	0.13
Night (vs twilight) ~ body size(medium)*feeding guild(herbivore)	0.40 (1.12)	0.36	0.72
Day (vs twilight) ~ body size(large)*feeding guild(omnivore)	-0.09 (1.68)	-0.05	0.96
Night (vs twilight) ~ body size(large)*feeding guild(omnivore)	1.43 (1.62)	0.88	0.38
Day (vs twilight) ~ body size(medium)*feeding guild(omnivore)	1.37 (1.23)	1.11	0.27
Night (vs twilight) ~ body size(medium)*feeding guild(omnivore)	0.20 (1.14)	0.17	0.86
Day (vs twilight) ~ disturbance*body size(large)*feeding guild(herbivore)	0.50 (0.22)	2.23	0.026*
Night (vs twilight) ~ disturbance*body size(large)*feeding guild(herbivore)	-0.54 (0.19)	-2.94	0.0033*
Day (vs twilight) ~ disturbance*body size(medium)*feeding guild(herbivore)	0.04 (0.10)	0.42	0.68
Night (vs twilight) ~ disturbance*body size(medium)*feeding guild(herbivore)	-0.09 (0.09)	-1.01	0.31
Day (vs twilight) ~ disturbance*body size(large)*feeding guild(omnivore)	0.39 (0.24)	1.61	0.11
Night (vs twilight) ~ disturbance*body size(large)*feeding guild(omnivore)	-0.28 (0.19)	-1.46	0.14
Day (vs twilight) ~ disturbance*body size(medium)*feeding guild(omnivore)	-0.10 (0.14)	-0.75	0.46
Night (vs twilight) ~ disturbance*body size(medium)*feeding guild(omnivore)	0.19 (0.11)	1.77	0.077

Supplementary Table 9. Multinomial logit model selection when only the 59 rarer specialists were considered. "Diel category" refers to species detections found within each diel category (*i.e.*, day, twilight, and night), "disturbance" is the inverse of the forest landscape integrity index (FLII), "body mass" is the mass of a species in kilograms, "body size" refers the body size categorisations given for each species [*i.e.*, large (>20 kg), medium (4-20 kg), and small (<4 kg)], and "feeding guild" is the typical diet of the species (*i.e.,* carnivore, herbivore, and omnivore).

**Model with landscape random effect**	**AICc**
Diel category ~ disturbance * body size * feeding guild	30676.86
Diel category ~ disturbance * body mass * feeding guild	31110.40
Diel category ~ disturbance + body size + feeding guild	31305.85
Diel category ~ disturbance * feeding guild	31314.29
Diel category ~ disturbance + feeding guild	31408.01
Diel category ~ feeding guild	31408.08
Diel category ~ disturbance + body mass + feeding guild	31411.99
Diel category ~ disturbance * body size	31653.50
Diel category ~ body size	31673.61
Diel category ~ disturbance + body size	31674.61
Diel category ~ disturbance * body mass	31731.84
Diel category ~ disturbance	31746.84
Diel category ~ body mass	31749.15
Diel category ~ disturbance + body mass	31750.06
**Model with landscape and species random effect**	**AICc**
Diel category ~ disturbance * body size	25949.77
Diel category ~ disturbance * body mass * feeding guild	25951.36
Diel category ~ disturbance * body mass	25952.07
Diel category ~ disturbance * body size * feeding guild	25953.44
Diel category ~ disturbance	25959.26
Diel category ~ feeding guild	25963.06
Diel category ~ body mass	25963.10

Diel category ~ disturbance + body mass	25963.18
Diel category ~ body size	25963.56
Diel category ~ disturbance * feeding guild	25965.21
Diel category ~ disturbance + feeding guild	25965.62
Diel category ~ disturbance + body size	25968.13
Diel category ~ disturbance + body mass + feeding guild	25968.33
Diel category ~ disturbance + body size + feeding guild	25969.18

Supplementary Table 10. Multinomial logit model summary testing for the effect of disturbance on the 59 rarer specialist species (excluding pigs and macaques) with only landscape considered as the random effect. The model selection showed the top model included a three-way interaction between disturbance, body size, and feeding guild (Table S9). The "twilight", "carnivore", and "small" were treated as the baseline or reference categories. Standard errors shown in parentheses and asterisks (*) denote significance ($P < 0.05$). Estimates of each model are obtained via the Restricted Maximum Likelihood (REML) method while p-values are estimated using the two-sided likelihood ratio test, however, no further statistical adjustments are made for each comparison.

Parameter	Estimates	Z-value	P-value
Day (vs twilight) ~ (Intercept)	-0.24 (0.16)	-1.54	0.12
Night (vs twilight) ~ (Intercept)	1.07 (0.14)	7.45	$9.3*10^{-14}$*
Day (vs twilight) ~ disturbance	0.04 (0.05)	0.88	0.38
Night (vs twilight) ~ disturbance	-0.08 (0.04)	-1.83	0.067
Day (vs twilight) ~ body size(large)	0.65 (0.38)	1.70	0.089
Night (vs twilight) ~ body size(large)	-2.36 (0.51)	-4.62	$3.8*10^{-6}$*
Day (vs twilight) ~ body size(medium)	-0.96 (0.24)	-3.98	$6.9*10^{-5}$*
Night (vs twilight) ~ body size(medium)	-0.46 (0.19)	-2.43	0.015*
Day (vs twilight) ~ feeding guild(herbivore)	0.23 (0.12)	1.89	0.059
Night (vs twilight) ~ feeding guild(herbivore)	-1.69 (0.11)	-15.50	$3.4*10^{-54}$*
Day (vs twilight) ~ feeding guild(omnivore)	-1.97 (0.33)	-5.92	$3.2*10^{-9}$*
Night (vs twilight) ~ feeding guild(omnivore)	0.62 (0.18)	3.35	$8.1*10^{-4}$*
Day (vs twilight) ~ disturbance*body size(large)	-0.36 (0.18)	-1.95	0.051
Night (vs twilight) ~ disturbance*body size(large)	0.39 (0.16)	2.40	0.017*
Day (vs twilight) ~ disturbance*body size(medium)	0.25 (0.07)	3.57	$3.5*10^{-4}$*
Night (vs twilight) ~ disturbance*body size(medium)	-0.08 (0.07)	-1.10	0.27
Day (vs twilight) ~ disturbance*feeding guild(herbivore)	-0.11 (0.05)	-2.30	0.022*
Night (vs twilight) ~ disturbance*feeding guild(herbivore)	0.11 (0.04)	2.57	0.010*
Day (vs twilight) ~ disturbance*feeding guild(omnivore)	0.19 (0.09)	2.09	0.037*
Night (vs twilight) ~ disturbance*feeding guild(omnivore)	-0.14 (0.07)	-2.06	0.039*

Day (vs twilight) ~ body size(large)*feeding guild(herbivore)	-0.95 (0.40)	-2.38	0.017*
Night (vs twilight) ~ body size(large)*feeding guild(herbivore)	3.25 (0.52)	6.25	$4.2*10^{-10}$*
Day (vs twilight) ~ body size(medium)*feeding guild(herbivore)	1.11 (0.25)	4.42	$1.0*10^{-7}$*
Night (vs twilight) ~ body size(medium)*feeding guild(herbivore)	1.25 (0.21)	6.07	$1.3*10^{-9}$*
Day (vs twilight) ~ body size(large)*feeding guild(omnivore)	1.40 (0.52)	2.69	0.0071*
Night (vs twilight) ~ body size(large)*feeding guild(omnivore)	0.64 (0.56)	1.14	0.25
Day (vs twilight) ~ body size(medium)*feeding guild(omnivore)	3.59 (0.45)	8.01	$1.2*10^{-15}$*
Night (vs twilight) ~ body size(medium)*feeding guild(omnivore)	-0.10 (0.31)	-0.33	0.75
Day (vs twilight) ~ disturbance*body size(large)*feeding guild(herbivore)	0.33 (0.19)	1.73	0.084
Night (vs twilight) ~ disturbance*body size(large)*feeding guild(herbivore)	-0.43 (0.17)	-2.58	0.010*
Day (vs twilight) ~ disturbance*body size(medium)*feeding guild(herbivore)	-0.15 (0.08)	-2.03	0.042*
Night (vs twilight) ~disturbance*body size(medium)*feeding guild(herbivore)	0.14 (0.08)	1.82	0.068
Day (vs twilight) ~ disturbance*body size(large)*feeding guild(omnivore)	0.17 (0.21)	0.81	0.42
Night (vs twilight) ~ disturbance*body size(large)*feeding guild(omnivore)	-0.16 (0.18)	-0.88	0.38
Day (vs twilight) ~ disturbance*body size(medium)*feeding guild(omnivore)	-0.71 (0.15)	-4.64	$3.4*10^{-6}$*
Night (vs twilight) ~ disturbance*body size(medium)*feeding guild(omnivore)	0.19 (0.12)	1.59	0.11

Supplementary Table 11. Multinomial logit model summary testing for the effect of disturbance on the 59 rarer specialist species (excluding pigs and macaques) while including both landscape and species-level random effect. The model selection showed the top model included a two-way interaction between disturbance and body size but not feeding guild (Table S9). The "twilight" and "small" were treated as the baseline or reference categories. Standard errors shown in parentheses and asterisks (*) denote significance ($P < 0.05$). Estimates of each model are obtained via the Restricted Maximum Likelihood (REML) method while p-values are estimated using the two-sided likelihood ratio test, however, no further statistical adjustments are made for each comparison.

Parameter	Estimates	Z-value	P-value
Day (vs twilight) ~ (Intercept)	-0.54	-1.58	0.11
Night (vs twilight) ~ (Intercept)	-0.42	-1.35	0.18
Day (vs twilight) ~ disturbance	-0.02	-0.82	0.41
Night (vs twilight) ~ disturbance	0.06	3.02	0.0025*
Day (vs twilight) ~ body size(large)	0.49	0.84	0.40
Night (vs twilight) ~ body size(large)	0.27	0.50	0.62
Day (vs twilight) ~ body size(medium)	0.28	0.59	0.56
Night (vs twilight) ~ body size(medium)	0.39	0.89	0.38
Day (vs twilight) ~ disturbance*body size(large)	-0.06	-1.54	0.12
Night (vs twilight) ~ disturbance*body size(large)	-0.02	-0.67	0.51
Day (vs twilight) ~ disturbance*body size(medium)	0.04	1.48	0.14
Night (vs twilight) ~ disturbance*body size(medium)	-0.09	-3.17	0.0015*

Supplementary Table 12. Multinomial logit model summary testing for the net effect of disturbance on all species. The "twilight" category was treated as the baseline or reference category in our analyses. Standard errors shown in parentheses and asterisks (*) denote significance ($P < 0.05$). Estimates of each model are obtained via the Restricted Maximum Likelihood (REML) method while p-values are estimated using the two-sided likelihood ratio test, however, no further statistical adjustments are made for each comparison.

**Parameters from models with landscape random effect**	**Estimates**	**Z-value**	**P-value**
Day (vs twilight) ~ (Intercept)	0.45 (0.07)	6.19	$6.0*10^{-10}$*
Night (vs twilight) ~ (Intercept)	-0.20 (0.14)	-1.50	0.13
Day (vs twilight) ~ disturbance	0.01 (0.01)	1.29	0.20
Night (vs twilight) ~ disturbance	-0.02 (0.01)	-1.61	0.11
**Parameters from models with landscape and species random effect**	**Estimates**	**Z-value**	**P-value**
Day (vs twilight) ~ (Intercept)	-0.28 (0.22)	-1.25	0.21
Night (vs twilight) ~ (Intercept)	-0.34 (0.20)	-1.77	0.077
Day (vs twilight) ~ disturbance	-0.01 (0.01)	-1.15	0.25
Night (vs twilight) ~ disturbance	0.03 (0.01)	2.35	0.019*

Supplementary Table 13. Multinomial logit model summary testing for the net effect of disturbance on the 59 rarer specialists. The "twilight" category was treated as the baseline or reference category in our analyses. Standard errors shown in parentheses and asterisks (*) denote significance ($P < 0.05$). Estimates of each model are obtained via the Restricted Maximum Likelihood (REML) method while p-values are estimated using the two-sided likelihood ratio test, however, no further statistical adjustments are made for each comparison.

**Parameters from models with landscape random effect**	**Estimates**	**Z-value**	**P-value**
Day (vs twilight) ~ (Intercept)	-0.06 (0.10)	-0.56	0.58
Night (vs twilight) ~ (Intercept)	0.16 (0.12)	1.34	0.18
Day (vs twilight) ~ disturbance	-0.03 (0.02)	-1.59	0.11
Night (vs twilight) ~ disturbance	0.002 (0.02)	0.11	0.91
**Parameters from models with landscape and species random effect**	**Estimates**	**Z-value**	**P-value**
Day (vs twilight) ~ (Intercept)	-0.35 (0.22)	-1.54	0.12
Night (vs twilight) ~ (Intercept)	-0.23 (0.21)	-1.08	0.28
Day (vs twilight) ~ disturbance	-0.008 (0.02)	-0.47	0.64
Night (vs twilight) ~ disturbance	0.03 (0.02)	1.75	0.080

Supplementary Table 14. Multinomial logit model summary testing for the net effect of disturbance on the four common pigs and macaques. The "twilight" category was treated as the baseline or reference category in our analyses. Standard errors shown in parentheses and asterisks (*) denote significance ($P < 0.05$). Estimates of each model are obtained via the Restricted Maximum Likelihood (REML) method while p-values are estimated using the two-sided likelihood ratio test, however, no further statistical adjustments are made for each comparison.

**Parameters from models with landscape random effect**	**Estimates**	**Z-value**	**P-value**
Day (vs twilight) ~ (Intercept)	0.96 (0.10)	9.30	$1.5*10^{-20}$*
Night (vs twilight) ~ (Intercept)	-1.28 (0.20)	-6.37	$1.8*10^{-10}$*
Day (vs twilight) ~ disturbance	-0.02 (0.01)	-1.64	0.10
Night (vs twilight) ~ disturbance	0.02 (0.02)	1.26	0.21
**Parameters from models with landscape and species random effect**	**Estimates**	**Z-value**	**P-value**
Day (vs twilight) ~ (Intercept)	1.03 (0.25)	4.22	$2.5*10^{-5}$*
Night (vs twilight) ~ (Intercept)	-1.48 (0.35)	-4.23	$2.3*10^{-5}$*
Day (vs twilight) ~ disturbance	-0.01 (0.01)	-1.29	0.20
Night (vs twilight) ~ disturbance	0.01 (0.02)	0.77	0.44

Supplementary Table 15. Binomial logit model summary testing for community-level wildlife behaviour shifts in response to disturbance (inverse FLII). These models compare the likelihood of detections occurring during the named period (left side of ~) versus detections occurring during any other period (compared to multinomial models that primarily compare against the reference category). In practice, for example, multinomial compares likelihood of detections during day vs twilight while the binomial below compares day versus either twilight or night. Both landscape and species were treated as the random effects. Standard errors are shown in parentheses and asterisks (*) denote significance ($P < 0.05$). Estimates of each model are obtained via the Restricted Maximum Likelihood (REML) method while p-values are estimated using the two-sided likelihood ratio test, however, no further statistical adjustments are made for each comparison.

Parameters	Estimates	Z-value	P-value
**All species**			
Day ~ (Intercept)	-1.02 (0.31)	-3.30	0.0010*
Day ~ disturbance	-0.02 (0.01)	-1.91	0.056
Night ~ (Intercept)	-1.07 (0.28)	-3.85	$1.2*10^{-4}$*
Night ~ disturbance	0.03 (0.01)	2.71	0.0068*
Twilight ~ (Intercept)	-1.04 (0.11)	-9.62	$6.8*10^{-22}$*
Twilight ~ disturbance	0.003 (0.1)	0.33	0.74
**Rarer 59 specialists**			
Day ~ (Intercept)	-1.13 (0.33)	-3.44	$5.8*10^{-4}$*
Day ~ disturbance	-0.02 (0.02)	-0.89	0.37
Night ~ (Intercept)	-0.93 (0.30)	-3.13	0.0017*
Night ~ disturbance	0.03 (0.02)	1.96	0.049*
Twilight ~ (Intercept)	-1.03 (0.11)	-9.38	$6.6*10^{-21}$*
Twilight ~ disturbance	-0.01 (0.01)	-1.10	0.27
**4 Pigs and macaques**			
Day ~ (Intercept)	0.79 (0.26)	3.05	0.0023*
Day ~ disturbance	-0.02 (0.01)	-1.53	0.12

Night ~ (Intercept)	-2.81 (0.43)	-6.46	$1.1*10^{-10}$*
Night ~ disturbance	0.02 (0.02)	1.35	0.18
Twilight ~ (Intercept)	-1.16 (0.19)	-6.24	$4.4*10^{-10}$*
Twilight ~ disturbance	0.01 (0.01)	1.11	0.27

Supplementary Table 16. Multinomial logit model summaries of guild-level responses to disturbance, with only landscape treated as random effects (no consideration of species-specific detections). The “twilight” was treated as the baseline or reference category. Standard errors shown in parentheses and asterisks (*) denote significance ($P < 0.05$). Estimates of each model are obtained via the Restricted Maximum Likelihood (REML) method while p-values are estimated using the two-sided likelihood ratio test, however, no further statistical adjustments are made for each comparison.

Trophic guild	Diel category	Estimates (SE)	Z-value	P-value
Large carnivore	Day (vs twilight) ~ (intercept)	0.89 (0.61)	1.46	0.14
	Night (vs twilight) ~ (intercept)	-1.77 (0.68)	-2.61	0.0090*
	Day (vs twilight) ~ disturbance	-0.53 (0.22)	-2.39	0.017*
	Night (vs twilight) ~ disturbance	0.42 (0.18)	2.36	0.018*
Large herbivore	Day (vs twilight) ~ (intercept)	-0.34 (0.15)	-2.23	0.026*
	Night (vs twilight) ~ (intercept)	0.02 (0.17)	0.09	0.93
	Day (vs twilight) ~ disturbance	-0.10 (0.05)	-1.95	0.051
	Night (vs twilight) ~ disturbance	0.02 (0.04)	0.38	0.70
Large omnivore	Day (vs twilight) ~ (intercept)	0.70 (0.22)	3.24	0.0012*
	Night (vs twilight) ~ (intercept)	-0.67 (0.22)	-3.06	0.0022*
	Day (vs twilight) ~ disturbance	-0.11 (0.02)	-7.01	$2.3*10^{-12}$*
	Night (vs twilight) ~ disturbance	-0.01 (0.02)	-0.40	0.69
Medium carnivore	Day (vs twilight) ~ (intercept)	-1.09 (0.40)	-2.72	0.0066*
	Night (vs twilight) ~ (intercept)	-0.03 (0.39)	-0.09	0.93
	Day (vs twilight) ~ disturbance	0.23 (0.08)	2.72	0.0066*
	Night (vs twilight) ~ disturbance	-0.01 (0.09)	-0.13	0.90
Medium herbivore	Day (vs twilight) ~ (intercept)	0.22 (0.16)	1.37	0.17
	Night (vs twilight) ~ (intercept)	0.45 (0.15)	3.05	0.0023*
	Day (vs twilight) ~ disturbance	-0.02 (0.03)	-0.68	0.50

	Night (vs twilight) ~ disturbance	0.03 (0.03)	1.01	0.31
Medium omnivore	Day (vs twilight) ~ (intercept)	1.00 (0.16)	6.34	$2.4*10^{-10}$*
	Night (vs twilight) ~ (intercept)	-1.59 (0.15)	-10.91	$1.1*10^{-27}$*
	Day (vs twilight) ~ disturbance	0.09 (0.02)	5.21	$1.9*10^{-7}$*
	Night (vs twilight) ~ disturbance	0.01 (0.03)	0.32	0.75
Small carnivore	Day (vs twilight) ~ (intercept)	-0.11 (0.18)	-0.60	0.55
	Night (vs twilight) ~ (intercept)	0.71 (0.18)	4.05	$5.1*10^{-5}$*
	Day (vs twilight) ~ disturbance	0.02 (0.06)	0.33	0.74
	Night (vs twilight) ~ disturbance	-0.02 (0.05)	-0.47	0.64
Small herbivore	Day (vs twilight) ~ (intercept)	-0.61 (0.25)	-2.43	0.015*
	Night (vs twilight) ~ (intercept)	-0.60 (0.30)	-2.01	0.044*
	Day (vs twilight) ~ disturbance	0.04 (0.03)	1.48	0.14
	Night (vs twilight) ~ disturbance	0.01 (0.03)	0.43	0.66
Small omnivore	Day (vs twilight) ~ (intercept)	-1.30 (0.51)	-2.55	0.011*
	Night (vs twilight) ~ (intercept)	1.38 (0.28)	4.94	$7.9*10^{-7}$*
	Day (vs twilight) ~ disturbance	0.04 (0.11)	0.40	0.69
	Night (vs twilight) ~ disturbance	-0.19 (0.07)	-2.73	0.0064

Supplementary Table 17. Multinomial logit model summaries of guild-level responses to disturbance, with both landscape and species were treated as random effects. Interpretations same as before.

Trophic guild	Diel category	Estimates (SE)	Z-value	P-value
Large carnivore	Day (vs twilight) ~ (intercept)	0.95 (0.72)	1.32	0.19
	Night (vs twilight) ~ (intercept)	-1.92 (0.77)	-2.51	0.012*
	Day (vs twilight) ~ disturbance	-0.51 (0.21)	-2.41	0.016*
	Night (vs twilight) ~ disturbance	0.43 (0.18)	2.42	0.016*
Large herbivore	Day (vs twilight) ~ (intercept)	-0.22 (0.42)	-0.53	0.60
	Night (vs twilight) ~ (intercept)	-0.06 (0.38)	-0.16	0.87
	Day (vs twilight) ~ disturbance	-0.04 (0.05)	-0.72	0.47
	Night (vs twilight) ~ disturbance	0.01 (0.05)	0.24	0.81
Large omnivore	Day (vs twilight) ~ (intercept)	0.35 (0.36)	0.98	0.33
	Night (vs twilight) ~ (intercept)	-0.43 (0.46)	-0.94	0.35
	Day (vs twilight) ~ disturbance	-0.12 (0.02)	-7.71	$1.3*10^{-14}$*
	Night (vs twilight) ~ disturbance	-0.01 (0.02)	-0.52	0.61
Medium carnivore	Day (vs twilight) ~ (intercept)	-0.79 (0.64)	-1.22	0.22
	Night (vs twilight) ~ (intercept)	-0.51 (0.51)	-0.99	0.32
	Day (vs twilight) ~ disturbance	0.11 (0.09)	1.26	0.21
	Night (vs twilight) ~ disturbance	0.03 (0.09)	0.33	0.74
Medium herbivore	Day (vs twilight) ~ (intercept)	0.56 (0.74)	0.76	0.45
	Night (vs twilight) ~ (intercept)	-0.66 (0.69)	-0.95	0.34
	Day (vs twilight) ~ disturbance	0.0005 (0.03)	0.02	0.99
	Night (vs twilight) ~ disturbance	-0.02 (0.03)	-0.77	0.44
Medium omnivore	Day (vs twilight) ~ (intercept)	-0.44 (0.67)	-0.65	0.51
	Night (vs twilight) ~ (intercept)	0.08 (0.61)	0.13	0.89
	Day (vs twilight) ~ disturbance	0.08 (0.02)	4.95	$7.6*10^{-7}$*
	Night (vs twilight) ~ disturbance	0.03 (0.03)	0.86	0.39
Small carnivore	Day (vs twilight) ~ (intercept)	-0.39 (0.57)	-0.68	0.50

	Night (vs twilight) ~ (intercept)	-0.13 (0.45)	-0.29	0.77
	Day (vs twilight) ~ disturbance	0.07 (0.06)	1.26	0.21
	Night (vs twilight) ~ disturbance	-0.0003 (0.05)	-0.08	0.94
Small herbivore	Day (vs twilight) ~ (intercept)	-1.02 (0.63)	-1.62	0.11
	Night (vs twilight) ~ (intercept)	-0.68 (0.77)	-0.88	0.38
	Day (vs twilight) ~ disturbance	0.03 (0.03)	1.00	0.32
	Night (vs twilight) ~ disturbance	0.08 (0.03)	2.69	0.0072*
Small omnivore	Day (vs twilight) ~ (intercept)	-1.03 (0.96)	-1.08	0.28
	Night (vs twilight) ~ (intercept)	-0.25 (1.07)	-0.24	0.81
	Day (vs twilight) ~ disturbance	0.11 (0.10)	1.05	0.29
	Night (vs twilight) ~ disturbance	-0.14 (0.05)	-2.50	0.012*

Supplementary Table 18. Model summaries for species-level multinomial logit models. All models were fitted by treating species detections found within each diel category (i.e., "day", "Twilight", and "Night) as the response variable and disturbance as the independent variable. We modelled and plotted FLII in decreasing order to showcase the effects of increasing disturbance on wildlife behaviour. We also included landscapes that we surveyed as a random effect in each model to account for non-independence. The "day" category was treated as the baseline or reference category in our analyses. Standard errors shown in parentheses and asterisks (*) denote significance (P < 0.05). Estimates of each model are obtained via the Restricted Maximum Likelihood (REML) method while p-values are estimated using the two-sided likelihood ratio test, however, no further statistical adjustments are made for each comparison.

Species	Diel category	Estimates (SE)	Z-value	P-value
Asian elephant (*Elephas maximus*)	Day (vs twilight) ~ (intercept)	0.42 (0.55)	0.77	0.44
	Night (vs twilight) ~ (intercept)	0.08 (0.45)	0.18	0.86
	Day (vs twilight) ~ disturbance	-0.64 (0.51)	-1.25	0.21
	Night (vs twilight) ~ disturbance	-0.23 (0.41)	-0.57	0.57
Banded civet (*Hemigalus derbyanus*)	Day (vs twilight) ~ (intercept)	-1.56 (0.31)	-5.02	$5.1*10^{-7}$*
	Night (vs twilight) ~ (intercept)	1.43 (0.15)	9.34	$9.5*10^{-21}$*
	Day (vs twilight) ~ disturbance	0.31 (0.16)	2.01	0.044*
	Night (vs twilight) ~ disturbance	0.07 (0.11)	0.60	0.55
Bearded pig (*Sus barbatus*)	Day (vs twilight) ~ (intercept)	0.60 (0.51)	1.17	0.24
	Night (vs twilight) ~ (intercept)	-1.23 (0.43)	-2.83	0.0046
	Day (vs twilight) ~ disturbance	-0.26 (0.03)	-7.41	$1.2*10^{-13}$*
	Night (vs twilight) ~ disturbance	0.05 (0.06)	0.83	0.40
Common long-tailed macaque (*Macaca fascicularis*)	Day (vs twilight) ~ (intercept)	3.08 (0.59)	5.24	$1.6*10^{-7}$*
	Night (vs twilight) ~ (intercept)	-1.74 (1.09)	-1.60	0.11
	Day (vs twilight) ~ disturbance	-0.21 (0.08)	-2.79	0.0053*
	Night (vs twilight) ~ disturbance	0.02 (0.14)	0.12	0.90

Great argus (*Argusianus argus*)	Day (vs twilight) ~ (intercept)	0.73 (0.09)	8.35	$6.9*10^{-17}$*
	Night (vs twilight) ~ (intercept)	-2.41 (0.26)	-9.21	$3.4*10^{-20}$*
	Day (vs twilight) ~ disturbance	-0.04 (0.04)	-1.04	0.30
	Night (vs twilight) ~ disturbance	-0.29 (0.29)	-1.02	0.31
Leopard cat (*Prionailurus bengalensis*)	Day (vs twilight) ~ (intercept)	-1.48 (0.33)	-4.47	$7.8*10^{-6}$*
	Night (vs twilight) ~ (intercept)	0.52 (0.22)	2.38	0.017*
	Day (vs twilight) ~ disturbance	0.11 (0.10)	1.13	0.26
	Night (vs twilight) ~ disturbance	-0.01 (0.07)	-0.14	0.89
Malay tapir (*Tapirus indicus*)	Day (vs twilight) ~ (intercept)	-1.40 (0.25)	-5.54	$3.0*10^{-8}$*
	Night (vs twilight) ~ (intercept)	0.48 (0.14)	3.45	$5.7*10^{-4}$*
	Day (vs twilight) ~ disturbance	-0.07 (0.08)	-0.86	0.39
	Night (vs twilight) ~ disturbance	-0.03 (0.04)	-0.74	0.46
Malayan porcupine (*Hystrix brachyura*)	Day (vs twilight) ~ (intercept)	-2.96 (0.34)	-8.71	$3.0*10^{-18}$*
	Night (vs twilight) ~ (intercept)	1.55 (0.09)	17.29	$5.9*10^{-67}$*
	Day (vs twilight) ~ disturbance	0.12 (0.10)	1.21	0.22
	Night (vs twilight) ~ disturbance	0.004 (0.03)	0.13	0.90
Mouse deer (*Tragulus spp.*)	Day (vs twilight) ~ (intercept)	-1.01 (0.22)	-4.50	$6.7*10^{-6}$*
	Night (vs twilight) ~ (intercept)	-1.04 (0.27)	-3.80	$1.5*10^{-4}$*
	Day (vs twilight) ~ disturbance	0.06 (0.03)	1.86	0.062
	Night (vs twilight) ~ disturbance	0.10 (0.03)	2.97	0.0029*
Sambar deer (*Rusa unicolor*)	Day (vs twilight) ~ (intercept)	-0.34 (0.23)	-1.50	0.13
	Night (vs twilight) ~ (intercept)	-0.05 (0.26)	-0.20	0.84
	Day (vs twilight) ~ disturbance	-0.02 (0.07)	-0.23	0.82
	Night (vs twilight) ~ disturbance	-0.02 (0.07)	-0.25	0.81
Pig-tailed macaque (*Macaca nemestrina*)	Day (vs twilight) ~ (intercept)	0.99 (0.20)	5.04	$4.6*10^{-7}$*
	Night (vs twilight) ~ (intercept)	-2.78 (0.34)	-8.14	$4.0*10^{-16}$*
	Day (vs twilight) ~ disturbance	0.10 (0.02)	5.79	$7.2*10^{-9}$*
	Night (vs twilight) ~ disturbance	0.05 (0.04)	1.15	0.25
Red muntjac (*Muntiacus muntjac*)	Day (vs twilight) ~ (intercept)	0.61 (0.14)	4.36	$1.3*10^{-5}$*

	Night (vs twilight) ~ (intercept)	-0.82 (0.16)	-5.01	$5.5*10^{-7}$*
	Day (vs twilight) ~ disturbance	0.0005 (0.03)	0.02	0.99
	Night (vs twilight) ~ disturbance	-0.04 (0.04)	-0.92	0.36
Sun bear (*Helarctos malayanus*)	Day (vs twilight) ~ (intercept)	-0.05 (0.26)	-0.20	0.84
	Night (vs twilight) ~ (intercept)	-0.48 (0.35)	-1.36	0.17
	Day (vs twilight) ~ disturbance	-0.07 (0.08)	-0.95	0.34
	Night (vs twilight) ~ disturbance	0.12 (0.08)	1.53	0.13
Tiger (*Panthera tigris*)	Day (vs twilight) ~ (intercept)	0.64 (0.57)	1.13	0.26
	Night (vs twilight) ~ (intercept)	-1.77 (0.60)	-2.98	0.0029*
	Day (vs twilight) ~ disturbance	-0.67 (0.32)	-2.10	0.036*
	Night (vs twilight) ~ disturbance	0.53 (0.21)	2.48	0.013*
Wild boar (*Sus scrofa*)	Day (vs twilight) ~ (intercept)	0.82 (0.16)	4.99	$6.0*10^{-7}$*
	Night (vs twilight) ~ (intercept)	-0.66 (0.26)	-2.55	0.011*
	Day (vs twilight) ~ disturbance	-0.07 (0.02)	-4.39	$1.1*10^{-5}$*
	Night (vs twilight) ~ disturbance	-0.02 (0.02)	-0.86	0.39

Supplementary Table 19. Species-level temporal outputs using kernel density estimation. 'AP_{Intact}' and '$AP_{Disturbed}$' signifies the activity peaks at intact (FLII > 8.77) and disturbed (FLII ≤ 8.77) sites respectively. P-values are estimated using the two-sided compareCkern test, however, no further statistical adjustments were made for each comparison (Note that the compareCkern function outputs "0" when P < 0.001, which we report here as "P < 0.001"). 'Δ (95% CI)' represents the estimated overlap coefficients and their associated 95% confidence interval. '1 - Δ' is the change in temporal overlap between intact and disturbed forests for a given species.

**Common name**	**Species name**	**P-value**	**AP_{Intact}**	**$AP_{Disturbed}$**	**Δ (95% CI)**	**1-Δ**
Asiatic brush-tailed porcupine	*Atherurus macrourus*	0.49	03:47 a.m.	02:03 a.m.	0.88 (0.80 – 0.95)	0.12
Banded civet	*Hemigalus derbyanus*	0.75	02:57 a.m.	00:50 a.m.	0.91 (0.84 – 0.98)	0.09
Banded linsang	*Prionodon linsang*	0.11	04:38 a.m.	03:36 a.m.	0.72 (0.57 – 0.86)	0.28
Bearded pig	*Sus barbatus*	<0.001	14:45 p.m.	17:12 p.m.	0.78 (0.73 – 0.83)	0.22
Bornean porcupine	*Hystrix crassispinis*	0.47	22:27 p.m.	21:36 p.m.	0.88 (0.78 – 0.98)	0.12
Clouded leopard	*Neofelis spp.*	0.008	22:07 p.m.	06:00 a.m.	0.72 (0.57 – 0.86)	0.28
Common palm civet	*Paradoxurus hermaphroditus*	0.094	19:38 p.m.	18:56 p.m.	0.84 (0.76 – 0.92)	0.16
Crested fireback pheasant	*Lophura spp.*	0.49	07:55 a.m.	08:23 a.m.	0.91 (0.86 – 0.97)	0.11
Great argus	*Argusianus argus*	<0.001	07:38 a.m.	06:56 a.m.	0.77 (0.68 – 0.86)	0.23
Leopard cat	*Prionailurus spp.*	0.019	05:17 a.m.	00:33 a.m.	0.84 (0.76 – 0.92)	0.16
Long-tailed porcupine	*Trichys fasciculata*	0.48	01:38 a.m.	20:09 p.m.	0.80 (0.65 – 0.95)	0.20
Malay tapir	*Tapirus indicus*	0.50	19:13 p.m.	19:38 p.m.	0.91 (0.84 – 0.98)	0.09
Malayan porcupine	*Hystrix brachyura*	0.31	01:52 a.m.	03:36 a.m.	0.95 (0.92 – 0.97)	0.05
Masked palm civet	*Paguma larvata*	0.80	20:12 p.m.	04:44 a.m.	0.86 (0.74 – 0.99)	0.14
Moon rat	*Echinosorex gymnura*	0.98	02:57 a.m.	02:51 a.m.	0.95 (0.89 – 1.01)	0.05
Mouse deer	*Tragulus spp.*	<0.001	06:14 a.m.	18:02 p.m.	0.89 (0.85 – 0.92)	0.11
Sambar deer	*Rusa unicolor*	0.21	06:16 a.m.	18:42 p.m.	0.89 (0.83 – 0.96)	0.11
Southern pig-tailed macaque	*Macaca nemestrina*	<0.001	08:23 a.m.	15:59 p.m.	0.87 (0.85 – 0.90)	0.13
Southern red muntjac	*Muntiacus muntjak*	<0.001	06:33 a.m.	07:18 a.m.	0.91 (0.89 – 0.94)	0.09
Sun bear	*Helarctos malayanus*	0.47	17:31 p.m.	17:43 p.m.	0.90 (0.84 – 0.96)	0.10
Tiger	*Panthera tigris*	0.002	06:36 a.m.	17:45 p.m.	0.63 (0.46 – 0.79)	0.37

Wild boar	*Sus scrofa*	<0.001	09:11 a.m.	06:53 a.m.	0.88 (0.86 – 0.90)	0.12
Yellow-throated marten	*Martes flavigula*	0.70	14:37 p.m.	06:59 a.m.	0.87 (0.76 – 0.97)	0.13

Supplementary Table 20. Estimated overlap coefficients (Δ) and their associated 95% confidence intervals (CI) between potential competitors in both intact (FLII > 8.77) and disturbed (FLII ≤ 8.77) sites across Southeast Asia. "*" refers to species pairs that are likely having a competitive interaction even though they have different body size categorisations (Refer to Methods section 2.7).

First species	Second species	Status	Competition type	Δ (95% CI)
Argusianus argus	*Atherurus macrourus*	Disturbed	herbivore-herbivore	0.13 (0.059 – 0.20)
Argusianus argus	*Atherurus macrourus*	Intact	herbivore-herbivore	0.097 (0.061 – 0.13)
Argusianus argus	*Lophura spp.*	Disturbed	herbivore-herbivore	0.83 (0.76 – 0.90)
Argusianus argus	*Lophura spp.*	Intact	herbivore-herbivore	0.79 (0.72 – 0.87)
Argusianus argus	*Tragulus spp.*	Disturbed	herbivore-herbivore	0.60 (0.53 – 0.67)
Argusianus argus	*Tragulus spp.*	Intact	herbivore-herbivore	0.58 (0.55 – 0.62)
Argusianus argus	*Trichys fasciculata*	Disturbed	herbivore-herbivore	0.13 (0.051 – 0.20)
Argusianus argus	*Trichys fasciculata*	Intact	herbivore-herbivore	0.097 (0.013 – 0.18)
**Atherurus macrourus*	**Hystrix brachyura*	Disturbed	herbivore-herbivore	0.89 (0.82 – 0.95)
**Atherurus macrourus*	**Hystrix brachyura*	Intact	herbivore-herbivore	0.95 (0.91 – 0.99)
Atherurus macrourus	*Lophura spp.*	Disturbed	herbivore-herbivore	0.080 (0.030 – 0.13)
Atherurus macrourus	*Lophura spp.*	Intact	herbivore-herbivore	0.079 (0.034 – 0.12)
Atherurus macrourus	*Tragulus spp.*	Disturbed	herbivore-herbivore	0.43 (0.36 – 0.49)
Atherurus macrourus	*Tragulus spp.*	Intact	herbivore-herbivore	0.35 (0.31 – 0.40)
Atherurus macrourus	*Trichys fasciculata*	Disturbed	herbivore-herbivore	0.73 (0.62 – 0.85)
Atherurus macrourus	*Trichys fasciculata*	Intact	herbivore-herbivore	0.87 (0.76 – 0.99)
Echinosorex gymnura	*Paradoxurus hermaphroditus*	Disturbed	omnivore-omnivore	0.72 (0.64 – 0.81)
Echinosorex gymnura	*Paradoxurus hermaphroditus*	Intact	omnivore-omnivore	0.83 (0.74 – 0.93)
Helarctos malayanus	*Sus barbatus*	Disturbed	omnivore-omnivore	0.73 (0.66 – 0.79)
Helarctos malayanus	*Sus barbatus*	Intact	omnivore-omnivore	0.71 (0.65 – 0.78)
Helarctos malayanus	*Sus scrofa*	Disturbed	omnivore-omnivore	0.75 (0.68 – 0.81)
Helarctos malayanus	*Sus scrofa*	Intact	omnivore-omnivore	0.67 (0.61 – 0.72)
Hemigalus derbyanus	*Martes flavigula*	Disturbed	carnivore-carnivore	0.26 (0.15 – 0.37)
Hemigalus derbyanus	*Martes flavigula*	Intact	carnivore-carnivore	0.17 (0.10 – 0.23)

Hemigalus derbyanus	*Prionailurus spp.*	Disturbed	carnivore-carnivore	0.83 (0.74 – 0.93)
Hemigalus derbyanus	*Prionailurus spp.*	Intact	carnivore-carnivore	0.82 (0.76 – 0.89)
Hemigalus derbyanus	*Prionodon linsang*	Disturbed	carnivore-carnivore	0.83 (0.72 – 0.95)
Hemigalus derbyanus	*Prionodon linsang*	Intact	carnivore-carnivore	0.70 (0.56 – 0.84)
Hystrix brachyura	*Hystrix crassispinis*	Disturbed	herbivore-herbivore	0.90 (0.82 – 0.97)
Hystrix brachyura	*Hystrix crassispinis*	Intact	herbivore-herbivore	0.82 (0.73 – 0.91)
Hystrix brachyura	*Muntiacus muntjak*	Disturbed	herbivore-herbivore	0.25 (0.22 – 0.28)
Hystrix brachyura	*Muntiacus muntjak*	Intact	herbivore-herbivore	0.29 (0.27 – 0.31)
**Hystrix brachyura*	**Trichys fasciculata*	Disturbed	herbivore-herbivore	0.83 (0.75 – 0.92)
**Hystrix brachyura*	**Trichys fasciculata*	Intact	herbivore-herbivore	0.90 (0.79 – 1.01)
Hystrix crassispinis	*Muntiacus muntjak*	Disturbed	herbivore-herbivore	0.26 (0.21 – 0.32)
Hystrix crassispinis	*Muntiacus muntjak*	Intact	herbivore-herbivore	0.34 (0.25 – 0.43)
**Hystrix crassispinis*	**Trichys fasciculata*	Disturbed	herbivore-herbivore	0.85 (0.75 – 0.94)
**Hystrix crassispinis*	**Trichys fasciculata*	Intact	herbivore-herbivore	0.83 (0.68 – 0.99)
Lophura spp.	*Tragulus spp.*	Disturbed	herbivore-herbivore	0.50 (0.45 – 0.54)
Lophura spp.	*Tragulus spp.*	Intact	herbivore-herbivore	0.60 (0.54 – 0.65)
Lophura spp.	*Trichys fasciculata*	Disturbed	herbivore-herbivore	0.13 (0.067 – 0.19)
Lophura spp.	*Trichys fasciculata*	Intact	herbivore-herbivore	0.085 (-0.0076 – 0.18)
Macaca nemestrina	*Paguma larvata*	Disturbed	omnivore-omnivore	0.19 (0.12 – 0.27)
Macaca nemestrina	*Paguma larvata*	Intact	omnivore-omnivore	0.20 (0.11 – 0.29)
Martes flavigula	*Prionailurus spp.*	Disturbed	carnivore-carnivore	0.41 (0.30 – 0.52)
Martes flavigula	*Prionailurus spp.*	Intact	carnivore-carnivore	0.27 (0.19 – 0.35)
Martes flavigula	*Prionodon linsang*	Disturbed	carnivore-carnivore	0.17 (0.063 – 0.28)
Martes flavigula	*Prionodon linsang*	Intact	carnivore-carnivore	0.20 (0.097 – 0.31)
**Muntiacus muntjak*	**Rusa unicolor*	Disturbed	herbivore-herbivore	0.66 (0.59 – 0.73)
**Muntiacus muntjak*	**Rusa unicolor*	Intact	herbivore-herbivore	0.81 (0.76 – 0.85)
**Muntiacus muntjak*	**Tapirus indicus*	Disturbed	herbivore-herbivore	0.47 (0.41 – 0.53)
**Muntiacus muntjak*	**Tapirus indicus*	Intact	herbivore-herbivore	0.49 (0.44 – 0.54)
**Muntiacus muntjak*	**Tragulus spp.*	Disturbed	herbivore-herbivore	0.71 (0.67 – 0.74)
**Muntiacus muntjak*	**Tragulus spp.*	Intact	herbivore-herbivore	0.81 (0.79 – 0.84)

**Neofelis spp.*	**Panthera tigris*	Disturbed	carnivore-carnivore	0.64 (0.48 – 0.80)
**Neofelis spp.*	**Panthera tigris*	Intact	carnivore-carnivore	0.44 (0.30 – 0.58)
Prionailurus spp.	*Prionodon linsang*	Disturbed	carnivore-carnivore	0.69 (0.57 – 0.81)
Prionailurus spp.	*Prionodon linsang*	Intact	carnivore-carnivore	0.78 (0.65 – 0.91)
Rusa unicolor	*Tapirus indicus*	Disturbed	herbivore-herbivore	0.80 (0.72 – 0.88)
Rusa unicolor	*Tapirus indicus*	Intact	herbivore-herbivore	0.69 (0.62 – 0.76)
**Rusa unicolor*	**Tragulus spp.*	Disturbed	herbivore-herbivore	0.82 (0.76 – 0.87)
**Rusa unicolor*	**Tragulus spp.*	Intact	herbivore-herbivore	0.82 (0.77 – 0.87)
Sus barbatus	*Sus scrofa*	Disturbed	omnivore-omnivore	0.82 (0.79 – 0.85)
Sus barbatus	*Sus scrofa*	Intact	omnivore-omnivore	0.90 (0.86 – 0.94)
**Tapirus indicus*	**Tragulus spp.*	Disturbed	herbivore-herbivore	0.69 (0.62 – 0.75)
**Tapirus indicus*	**Tragulus spp.*	Intact	herbivore-herbivore	0.59 (0.54 – 0.65)
Tragulus spp.	*Trichys fasciculata*	Disturbed	herbivore-herbivore	0.49 (0.41 – 0.56)
Tragulus spp.	*Trichys fasciculata*	Intact	herbivore-herbivore	0.33 (0.21 – 0.44)

Supplementary Table 21. Mann-Whitney U test outputs at the guild-level for potential competitors between intact (FLII > 8.77) and disturbed (FLII ≤ 8.77) forests. 'N' refers to the number of species pairs used for the analysis. We also note that the statistical tests used are two-sided and no further statistical adjustments were conducted for each pairwise comparisons.

Competition type	$N_{Disturbed}$	N_{Intact}	$Mean_{Intact}$ (95%CI)	$Mean_{Disturbed}$ (95%CI)	U-statistics	P-value
carnivore-carnivore	7	7	0.48 (0.29 – 0.70)	0.55 (0.35 – 0.72)	4	0.11
herbivore-herbivore	22	22	0.56 (0.43 – 0.68)	0.55 (0.43 – 0.66)	125	0.97
omnivore-omnivore	5	5	0.66 (0.43 – 0.84)	0.64 (0.41 – 0.78)	9	0.81

Supplementary Table 22. Estimated overlap coefficients (Δ) and their associated 95% confidence intervals (CI) between potential predators and prey in both intact (FLII > 8.77) and disturbed (FLII ≤ 8.77) sites across Southeast Asia. Species pairs involving clouded leopards (*Neofelis spp.*) are given exceptions ("*") as the species is able to predate upon other species with a larger body size category than itself (Refer to Methods section 2.7).

First species	Second species	Status	Predation type	Δ (95% CI)
Argusianus argus	*Hemigalus derbyanus*	Disturbed	carnivore-herbivore	0.21 (0.11 – 0.30)
Argusianus argus	*Hemigalus derbyanus*	Intact	carnivore-herbivore	0.14 (0.10 – 0.17)
Argusianus argus	*Martes flavigula*	Disturbed	carnivore-herbivore	0.91 (0.82 – 0.99)
Argusianus argus	*Martes flavigula*	Intact	carnivore-herbivore	0.75 (0.65 – 0.84)
Argusianus argus	*Neofelis spp.*	Disturbed	carnivore-herbivore	0.39 (0.28 – 0.51)
Argusianus argus	*Neofelis spp.*	Intact	carnivore-herbivore	0.26 (0.18 – 0.34)
Argusianus argus	*Panthera tigris*	Disturbed	carnivore-herbivore	0.54 (0.39 – 0.68)
Argusianus argus	*Panthera tigris*	Intact	carnivore-herbivore	0.72 (0.61 – 0.83)
Argusianus argus	*Prionailurus spp.*	Disturbed	carnivore-herbivore	0.37 (0.27 – 0.46)
Argusianus argus	*Prionailurus spp.*	Intact	carnivore-herbivore	0.26 (0.20 – 0.31)
Argusianus argus	*Prionodon linsang*	Disturbed	carnivore-herbivore	0.13 (0.045 – 0.22)
Argusianus argus	*Prionodon linsang*	Intact	carnivore-herbivore	0.20 (0.10 – 0.29)
Atherurus macrourus	*Hemigalus derbyanus*	Disturbed	carnivore-herbivore	0.84 (0.75 – 0.93)
Atherurus macrourus	*Hemigalus derbyanus*	Intact	carnivore-herbivore	0.92 (0.87 – 0.98)
Atherurus macrourus	*Martes flavigula*	Disturbed	carnivore-herbivore	0.17 (0.078 – 0.26)
Atherurus macrourus	*Martes flavigula*	Intact	carnivore-herbivore	0.13 (0.063 – 0.19)
Atherurus macrourus	*Neofelis spp.*	Disturbed	carnivore-herbivore	0.64 (0.50 – 0.76)
Atherurus macrourus	*Neofelis spp.*	Intact	carnivore-herbivore	0.84 (0.76 – 0.91)

Atherurus macrourus	*Panthera tigris*	Disturbed	carnivore-herbivore	0.53 (0.38 – 0.67)
Atherurus macrourus	*Panthera tigris*	Intact	carnivore-herbivore	0.29 (0.16 – 0.41)
Atherurus macrourus	*Prionailurus spp.*	Disturbed	carnivore-herbivore	0.70 (0.60 – 0.79)
Atherurus macrourus	*Prionailurus spp.*	Intact	carnivore-herbivore	0.81 (0.75 – 0.88)
Atherurus macrourus	*Prionodon linsang*	Disturbed	carnivore-herbivore	0.79 (0.66 – 0.92)
Atherurus macrourus	*Prionodon linsang*	Intact	carnivore-herbivore	0.73 (0.59 – 0.86)
Echinosorex gymnura	*Hemigalus derbyanus*	Disturbed	carnivore-omnivore	0.87 (0.79 – 0.95)
Echinosorex gymnura	*Hemigalus derbyanus*	Intact	carnivore-omnivore	0.87 (0.80 – 0.94)
Echinosorex gymnura	*Martes flavigula*	Disturbed	carnivore-omnivore	0.15 (0.069 – 0.24)
Echinosorex gymnura	*Martes flavigula*	Intact	carnivore-omnivore	0.11 (0.043 – 0.17)
Echinosorex gymnura	*Neofelis spp.*	Disturbed	carnivore-omnivore	0.63 (0.50 – 0.76)
Echinosorex gymnura	*Neofelis spp.*	Intact	carnivore-omnivore	0.77 (0.69 – 0.86)
Echinosorex gymnura	*Panthera tigris*	Disturbed	carnivore-omnivore	0.52 (0.38 – 0.66)
Echinosorex gymnura	*Panthera tigris*	Intact	carnivore-omnivore	0.26 (0.14 – 0.39)
Echinosorex gymnura	*Prionailurus spp.*	Disturbed	carnivore-omnivore	0.71 (0.62 – 0.79)
Echinosorex gymnura	*Prionailurus spp.*	Intact	carnivore-omnivore	0.76 (0.69 – 0.83)
Echinosorex gymnura	*Prionodon linsang*	Disturbed	carnivore-omnivore	0.86 (0.74 – 0.98)
Echinosorex gymnura	*Prionodon linsang*	Intact	carnivore-omnivore	0.69 (0.56 – 0.83)
**Helarctos malayanus*	**Neofelis spp.*	Disturbed	carnivore-omnivore	0.60 (0.48 – 0.72)
**Helarctos malayanus*	**Neofelis spp.*	Intact	carnivore-omnivore	0.62 (0.53 – 0.72)
Helarctos malayanus	*Panthera tigris*	Disturbed	carnivore-omnivore	0.75 (0.62 – 0.88)
Helarctos malayanus	*Panthera tigris*	Intact	carnivore-omnivore	0.69 (0.56 – 0.81)
Hemigalus derbyanus	*Lophura spp.*	Disturbed	carnivore-herbivore	0.18 (0.10 – 0.25)
Hemigalus derbyanus	*Lophura spp.*	Intact	carnivore-herbivore	0.13 (0.083 – 0.17)
Hemigalus derbyanus	*Neofelis spp.*	Disturbed	carnivore-carnivore	0.68 (0.54 – 0.82)
Hemigalus derbyanus	*Neofelis spp.*	Intact	carnivore-carnivore	0.85 (0.78 – 0.93)

Hemigalus derbyanus	*Panthera tigris*	Disturbed	carnivore-carnivore	0.65 (0.51 – 0.79)
Hemigalus derbyanus	*Panthera tigris*	Intact	carnivore-carnivore	0.32 (0.19 – 0.44)
Hemigalus derbyanus	*Paradoxurus hermaphroditus*	Disturbed	carnivore-carnivore	0.80 (0.71 – 0.89)
Hemigalus derbyanus	*Paradoxurus hermaphroditus*	Intact	carnivore-carnivore	0.86 (0.76 – 0.93)
Hemigalus derbyanus	*Tragulus spp.*	Disturbed	carnivore-carnivore	0.53 (0.45 – 0.62)
Hemigalus derbyanus	*Tragulus spp.*	Intact	carnivore-carnivore	0.40 (0.35 – 0.44)
Hemigalus derbyanus	*Trichys fasciculata*	Disturbed	carnivore-herbivore	0.82 (0.71 – 0.93)
Hemigalus derbyanus	*Trichys fasciculata*	Intact	carnivore-herbivore	0.89 (0.77 – 1.00)
Hystrix brachyura	*Neofelis spp.*	Disturbed	carnivore-herbivore	0.64 (0.51 – 0.77)
Hystrix brachyura	*Neofelis spp.*	Intact	carnivore-herbivore	0.83 (0.76 – 0.90)
Hystrix brachyura	*Panthera tigris*	Disturbed	carnivore-herbivore	0.55 (0.42 – 0.68)
Hystrix brachyura	*Panthera tigris*	Intact	carnivore-herbivore	0.27 (0.15 – 0.39)
Hystrix crassispinis	*Neofelis spp.*	Disturbed	carnivore-herbivore	0.59 (0.45 – 0.74)
Hystrix crassispinis	*Neofelis spp.*	Intact	carnivore-herbivore	0.80 (0.68 – 0.92)
Lophura spp.	*Martes flavigula*	Disturbed	carnivore-herbivore	0.80 (0.71 – 0.89)
Lophura spp.	*Martes flavigula*	Intact	carnivore-herbivore	0.80 (0.71 – 0.89)
Lophura spp.	*Neofelis spp.*	Disturbed	carnivore-herbivore	0.26 (0.16 – 0.36)
Lophura spp.	*Neofelis spp.*	Intact	carnivore-herbivore	0.25 (0.16 – 0.33)
Lophura spp.	*Panthera tigris*	Disturbed	carnivore-herbivore	0.48 (0.34 – 0.62)
Lophura spp.	*Panthera tigris*	Intact	carnivore-herbivore	0.70 (0.58 – 0.82)
Lophura spp.	*Prionailurus spp.*	Disturbed	carnivore-herbivore	0.32 (0.24 – 0.40)
Lophura spp.	*Prionailurus spp.*	Intact	carnivore-herbivore	0.25 (0.19 – 0.32)
Lophura spp.	*Prionodon linsang*	Disturbed	carnivore-herbivore	0.10 (0.026 – 0.17)
Lophura spp.	*Prionodon linsang*	Intact	carnivore-herbivore	0.19 (0.087 – 0.29)
Macaca nemestrina	*Neofelis spp.*	Disturbed	carnivore-omnivore	0.35 (0.26 – 0.44)
Macaca nemestrina	*Neofelis spp.*	Intact	carnivore-omnivore	0.22 (0.15 – 0.30)

Macaca nemestrina	*Panthera tigris*	Disturbed	carnivore-omnivore	0.53 (0.39 – 0.66)
Macaca nemestrina	*Panthera tigris*	Intact	carnivore-omnivore	0.66 (0.54 – 0.79)
Martes flavigula	*Neofelis spp.*	Disturbed	carnivore-carnivore	0.43 (0.30 – 0.56)
Martes flavigula	*Neofelis spp.*	Intact	carnivore-carnivore	0.27 (0.17 – 0.37)
Martes flavigula	*Panthera tigris*	Disturbed	carnivore-carnivore	0.55 (0.40 – 0.70)
Martes flavigula	*Panthera tigris*	Intact	carnivore-carnivore	0.69 (0.55 – 0.83)
Martes flavigula	*Paradoxurus hermaphroditus*	Disturbed	carnivore-omnivore	0.27 (0.17 – 0.37)
Martes flavigula	*Paradoxurus hermaphroditus*	Intact	carnivore-omnivore	0.14 (0.068 – 0.21)
Martes flavigula	*Tragulus spp.*	Disturbed	carnivore-herbivore	0.60 (0.51 – 0.69)
Martes flavigula	*Tragulus spp.*	Intact	carnivore-herbivore	0.56 (0.48 – 0.64)
Martes flavigula	*Trichys fasciculata*	Disturbed	carnivore-herbivore	0.18 (0.080 – 0.27)
Martes flavigula	*Trichys fasciculata*	Intact	carnivore-herbivore	0.11 (0.012 – 0.21)
Muntiacus muntjak	*Neofelis spp.*	Disturbed	carnivore-herbivore	0.51 (0.40 – 0.61)
Muntiacus muntjak	*Neofelis spp.*	Intact	carnivore-herbivore	0.46 (0.38 – 0.55)
Muntiacus muntjak	*Panthera tigris*	Disturbed	carnivore-herbivore	0.66 (0.54 – 0.79)
Muntiacus muntjak	*Panthera tigris*	Intact	carnivore-herbivore	0.82 (0.73 – 0.91)
Neofelis spp.	*Paguma larvata*	Disturbed	carnivore-omnivore	0.76 (0.62 – 0.91)
Neofelis spp.	*Paguma larvata*	Intact	carnivore-omnivore	0.87 (0.76 – 0.98)
Neofelis spp.	*Paradoxurus hermaphroditus*	Disturbed	carnivore-omnivore	0.71 (0.59 – 0.83)
Neofelis spp.	*Paradoxurus hermaphroditus*	Intact	carnivore-omnivore	0.82 (0.73 – 0.91)
Neofelis spp.	*Prionodon linsang*	Disturbed	carnivore-omnivore	0.67 (0.52 – 0.81)
Neofelis spp.	*Prionodon linsang*	Intact	carnivore-omnivore	0.71 (0.73 – 0.91)
**Neofelis spp.*	**Rusa unicolor*	Disturbed	carnivore-herbivore	0.67 (0.56 – 0.86)
**Neofelis spp.*	**Rusa unicolor*	Intact	carnivore-herbivore	0.63 (0.54 – 0.73)
**Neofelis spp.*	**Sus barbatus*	Disturbed	carnivore-omnivore	0.46 (0.35 – 0.56)
**Neofelis spp.*	**Sus barbatus*	Intact	carnivore-omnivore	0.39 (0.30 – 0.47)

**Neofelis spp.*	**Sus scrofa*	Disturbed	carnivore-omnivore	0.50 (0.41 – 0.59)
**Neofelis spp.*	**Sus scrofa*	Intact	carnivore-omnivore	0.37 (0.29 – 0.44)
**Neofelis spp.*	**Tapirus indicus*	Disturbed	carnivore-herbivore	0.74 (0.61 – 0.87)
**Neofelis spp.*	**Tapirus indicus*	Intact	carnivore-herbivore	0.83 (0.75 – 0.92)
Neofelis spp.	*Tragulus spp.*	Disturbed	carnivore-herbivore	0.67 (0.57 – 0.77)
Neofelis spp.	*Tragulus spp.*	Intact	carnivore-herbivore	0.52 (0.43 – 0.61)
Neofelis spp.	*Trichys fasciculata*	Disturbed	carnivore-herbivore	0.55 (0.41 – 0.69)
Neofelis spp.	*Trichys fasciculata*	Intact	carnivore-herbivore	0.80 (0.66 – 0.94)
Paguma larvata	*Panthera tigris*	Disturbed	carnivore-omnivore	0.63 (0.49 – 0.78)
Paguma larvata	*Panthera tigris*	Intact	carnivore-omnivore	0.44 (0.28 – 0.59)
Panthera tigris	*Paradoxurus hermaphroditus*	Disturbed	carnivore-omnivore	0.70 (0.57 – 0.82)
Panthera tigris	*Paradoxurus hermaphroditus*	Intact	carnivore-omnivore	0.30 (0.17 – 0.43)
Panthera tigris	*Prionailurus spp.*	Disturbed	carnivore-carnivore	0.75 (0.63 – 0.88)
Panthera tigris	*Prionailurus spp.*	Intact	carnivore-carnivore	0.46 (0.33 – 0.60)
Panthera tigris	*Prionodon linsang*	Disturbed	carnivore-carnivore	0.56 (0.42 – 0.71)
Panthera tigris	*Prionodon linsang*	Intact	carnivore-carnivore	0.40 (0.25 – 0.55)
Panthera tigris	*Rusa unicolor*	Disturbed	carnivore-herbivore	0.85 (0.74 – 0.96)
Panthera tigris	*Rusa unicolor*	Intact	carnivore-herbivore	0.73 (0.61 – 0.85)
Panthera tigris	*Sus barbatus*	Disturbed	carnivore-omnivore	0.62 (0.50 – 0.74)
Panthera tigris	*Sus barbatus*	Intact	carnivore-omnivore	0.75 (0.62 – 0.88)
Panthera tigris	*Sus scrofa*	Disturbed	carnivore-omnivore	0.70 (0.57 – 0.83)
Panthera tigris	*Sus scrofa*	Intact	carnivore-omnivore	0.78 (0.65 – 0.90)
Panthera tigris	*Tapirus indicus*	Disturbed	carnivore-herbivore	0.75 (0.63 – 0.88)
Panthera tigris	*Tapirus indicus*	Intact	carnivore-herbivore	0.45 (0.32 – 0.58)
Panthera tigris	*Tragulus spp.*	Disturbed	carnivore-herbivore	0.75 (0.64 – 0.86)
Panthera tigris	*Tragulus spp.*	Intact	carnivore-herbivore	0.74 (0.64 – 0.84)

Panthera tigris	*Trichys fasciculata*	Disturbed	carnivore-herbivore	0.58 (0.44 – 0.72)
Panthera tigris	*Trichys fasciculata*	Intact	carnivore-herbivore	0.25 (0.098 – 0.40)
Paradoxurus hermaphroditus	*Prionailurus spp.*	Disturbed	carnivore-herbivore	0.77 (0.68 – 0.86)
Paradoxurus hermaphroditus	*Prionailurus spp.*	Intact	carnivore-herbivore	0.83 (0.75 – 0.91)
Paradoxurus hermaphroditus	*Prionodon linsang*	Disturbed	carnivore-herbivore	0.77 (0.67 – 0.87)
Paradoxurus hermaphroditus	*Prionodon linsang*	Intact	carnivore-herbivore	0.73 (0.59 – 0.88)
Prionailurus spp.	*Tragulus spp.*	Disturbed	carnivore-herbivore	0.71 (0.63 – 0.78)
Prionailurus spp.	*Tragulus spp.*	Intact	carnivore-herbivore	0.56 (0.49 – 0.62)
Prionailurus spp.	*Trichys fasciculata*	Disturbed	carnivore-herbivore	0.75 (0.66 – 0.84)
Prionailurus spp.	*Trichys fasciculata*	Intact	carnivore-herbivore	0.74 (0.60 – 0.87)
Prionodon linsang	*Tragulus spp.*	Disturbed	carnivore-herbivore	0.47 (0.37 – 0.56)
Prionodon linsang	*Tragulus spp.*	Intact	carnivore-herbivore	0.50 (0.38 – 0.61)
Prionodon linsang	*Trichys fasciculata*	Disturbed	carnivore-herbivore	0.79 (0.65 – 0.92)
Prionodon linsang	*Trichys fasciculata*	Intact	carnivore-herbivore	0.62 (0.44 – 0.80)

Supplementary Table 23. Mann-Whitney U test outputs at the guild-level for potential predatory interactions between intact (FLII > 8.77) and disturbed (FLII ≤ 8.77) forests. 'N' refers to the number of species pairs used for the analysis. We also note that the statistical tests used are two-sided and no further statistical adjustments were conducted for each pairwise comparisons.

Predation type	$N_{Disturbed}$	N_{Intact}	$Mean_{Intact}$ (95%CI)	$Mean_{Disturbed}$ (95%CI)	U-statistics	P-value
carnivore-carnivore	8	8	0.58 (0.42 – 0.74)	0.62 (0.55 – 0.68)	14	0.64
carnivore-herbivore	39	39	0.53 (0.45 – 0.62)	0.55 (0.45 – 0.61)	331	0.42
carnivore-omnivore	22	22	0.59 (0.48 – 0.69)	0.62 (0.54 – 0.69)	100	0.41
