# Supplementary material for: Effects of human disturbances on wildlife behaviour and consequences for predator-prey overlap in Southeast Asia
Source: Nat Commun. 2024 Feb 19;15:1521. doi: 10.1038/s41467-024-45905-9 (PMC10876642; doi:10.1038/s41467-024-45905-9)
Supplement: Supplementary file 3 — Reporting Summary [file 41467_2024_45905_MOESM3_ESM.pdf]

nature portfolio

Corresponding author(s): Matthew Scott Luskin

Last updated by author(s): Jan 25, 2024

Reporting Summary

Nature Portfolio wishes to improve the reproducibility of the work that we publish. This form provides structure for consistency and transparency in reporting. For further information on Nature Portfolio policies, see our Editorial Policies and the Editorial Policy Checklist.

Statistics

For all statistical analyses, confirm that the following items are present in the figure legend, table legend, main text, or Methods section.

n/a	Confirmed	
☐	☒	The exact sample size (*n*) for each experimental group/condition, given as a discrete number and unit of measurement
☒	☐	A statement on whether measurements were taken from distinct samples or whether the same sample was measured repeatedly
☐	☒	The statistical test(s) used AND whether they are one- or two-sided<br>*Only common tests should be described solely by name; describe more complex techniques in the Methods section.*
☐	☒	A description of all covariates tested
☒	☐	A description of any assumptions or corrections, such as tests of normality and adjustment for multiple comparisons
☐	☒	A full description of the statistical parameters including central tendency (e.g. means) or other basic estimates (e.g. regression coefficient) AND variation (e.g. standard deviation) or associated estimates of uncertainty (e.g. confidence intervals)
☐	☒	For null hypothesis testing, the test statistic (e.g. *F*, *t*, *r*) with confidence intervals, effect sizes, degrees of freedom and *P* value noted<br>*Give P values as exact values whenever suitable.*
☒	☐	For Bayesian analysis, information on the choice of priors and Markov chain Monte Carlo settings
☒	☐	For hierarchical and complex designs, identification of the appropriate level for tests and full reporting of outcomes
☒	☐	Estimates of effect sizes (e.g. Cohen's *d*, Pearson's *r*), indicating how they were calculated

Our web collection on statistics for biologists contains articles on many of the points above.

Software and code

Policy information about availability of computer code

Data collection	We sample wildlife with camera traps at 10 landscapes in Thailand, Peninsular Malaysia, Singapore, Sumatra, and Malaysian Borneo between 2013 to 2019. We deploy 18-78 passive infrared Bushnell and Reconyx cameras for 60–90 days covering 10 to 813 km² at each landscape. We standardise deployment methods across all landscapes by spacing cameras >500 m in larger forests (>50 km²) and 100–500 m apart in smaller forest patches (e.g., Singapore) and attaching them to trees 0.3 m above ground along natural wildlife trails or hiking paths. We also extracted species trait data from the Pantheria database (mammals), Avonet Database (birds), and other published works (reptiles). References for the databases and published work are included in the main text. The raw species captures and the standardized species captures can be obtain via Figshare using this link: ttps://doi.org/10.6084/m9.figshare.23513412.
Data analysis	We calculated activity distributions with circular kernel probability functions and 95% confidence intervals (CIs) with the fitact() function in the ‘activity’ package version 1.3.3 (bootstrapping 10,000 iterations). We calculated the coefficient of overlap (Δ) between activity distributions using the OverlapEST() function in the ‘overlap’ package version 0.3.4. Δ denotes the shared area under the two activity distributions and ranges from 0 (no overlap) to 1 (full overlap). Following Ridout and Linkie, we report the Δ4 overlap estimator with a smoothing parameter of 1 when the sample size iswas >75 and the Δ1 overlap estimator with a smoothing parameter of 0.8 when the sample size is ≤75. We used the compareCkern() function found in the ‘activity’ package to assess the statistical significance between activity distributions. This function is a randomization test that generates a null distribution of overlap indices using data sampled randomly with replacement from the combined datasets. We also determined the activity peaks (AP) within each diel categories [i.e., day (0730 – 1630 hrs; 9 hours total), twilight (0430 – 0730 hrs or 1630 – 1930 hrs; 6 hours total) and night (1930 – 0430 hrs; 9 hours total)] for each species, guild, and community, using the highest densities for each activity distribution. We then evaluated biologically meaningful behavioural shifts using two criteria: (1) a change in activity peak between day, twilight, or night, and (2) a p-value of < 0.05 from the circular distribution randomisation test comparing the activity distributions in intact and disturbed forests. These two criteria account for either statistically significant and/or large shifts in activity peak within a single period (e.g., from 0900 h to 1530 h is a large shift within the same diurnal period) or small activity shifts that cross

threshold among day, twilight and night diel categories (e.g., a change in peak activity from 0735 h to 0725 h).

We use multinomial logit mixed models (MNLMMs) with three response variable categories (i.e., day, twilight, and night) and random effects to assess the probability of wildlife detections in each diel category in response to disturbance. We fit community-, guild-, and species-level models and use forest integrity as our disturbance covariate. For our community-level models, we also add two other covariates of interests, such as body size (i.e., large, medium, and small), body mass (in kg) and feeding guild (i.e., carnivore, herbivore, and omnivore) as well as their interactions with forest integrity in mediating diel responses to disturbance. For each community-, guild-, and species-level models, we calculate the Akaike Information Criterion (AIC) score to select the best model (i.e., model with the lowest AIC score) for each animal grouping. We treat landscape as a random effect for all our models and ran all our models using restricted maximum likelihood (REML). To test for behavioural adaptations across the disturbance gradient, we also include species-level random effects in the main text results, where species are weighted similarly regardless of differences in the number of detections. To test how species turnover affected community- and guild-level results, we remove the species random effect thereby allowing detections to be weighted equally regardless of species which is shown in the supplementary materials. We set twilight as the reference category for all our models. We implement all MNLMMs in the 'mclogit' package in R and plot the predicted probabilities for each diel category using the package "stats" version 4.3.1.

To understand the change in species overlap within disturbed forests, we first extracted activity distributions and Δ overlap for all competitor pairs and pairs of predators and their potential prey. We define species pairs as having potential 'competitive interactions' when species exhibit overlapping ranges and share the same body size and feeding category. Exceptions include all sizes of porcupines (both small and medium-sized), which are allowed to compete, and all sizes of strictly herbivorous ungulates [i.e., Malay tapir (Tapirus Indicus), sambar deer (Rusa unicolor), red muntjac (Muntiacus muntjak), and mouse deer], which are also allowed to compete. We defined species pairs as having potential 'competitive interactions' when species have overlapping ranges and have the same body size and feeding category, with the exceptions of all porcupines (both small and medium-sized) that were allowed to compete and all sizes of strictly herbivorous ungulates were allowed to compete [i.e., Malay tapir (Tapirus Indicus), sambar deer (Rusa unicolor), red muntjac (Muntiacus muntjak) and mouse deer]. For apex predators, we allowed tiger, leopard, and clouded leopards to compete because they are known to share prey species. Predator-prey pairs were are established for species with (i) overlapping ranges, (ii) at least one or both species possessing a predominantly carnivorous diet, and (iii) predators could can only predate species of the same size category or lower (with an exception for medium-sized clouded leopards that were are allowed to predate large ungulates, as noted above). We also assumed that omnivores do not predate other species, since most omnivorous species within our community were primarily included in this grouping because they consume insects, fungi, carrion, and potentially animals <1 kg such as rodents and birds that were are excluded from this study. We then calculated the species-level pairwise overlap in disturbed and intact forests and used a paired t-test to determine significant differences. Lastly, we used the Mann-Whitney test when species pairs were are further split into their respective guilds and similarly determine significant differences between forest types. We conducted all our analyses using the R statistical software version 4.2.0.

All packages used are described fully and referenced in the main text. The code scripts used for these analyses can be accessed via GitHub using this link: https://github.com/EcologicalCascadesLab/WildlifeActivityPatterns

For manuscripts utilizing custom algorithms or software that are central to the research but not yet described in published literature, software must be made available to editors and reviewers. We strongly encourage code deposition in a community repository (e.g. GitHub). See the Nature Portfolio guidelines for submitting code & software for further information.

Data

Policy information about availability of data

All manuscripts must include a data availability statement. This statement should provide the following information, where applicable:

- Accession codes, unique identifiers, or web links for publicly available datasets
- A description of any restrictions on data availability
- For clinical datasets or third party data, please ensure that the statement adheres to our policy

The full camera trapping and species trait data collected for this study can be accessed via Figshare using the following DOI: https://doi.org/10.6084/m9.figshare.23513412.

Research involving human participants, their data, or biological material

Policy information about studies with human participants or human data. See also policy information about sex, gender (identity/presentation), and sexual orientation and race, ethnicity and racism.

Reporting on sex and gender	NA
Reporting on race, ethnicity, or other socially relevant groupings	NA
Population characteristics	NA
Recruitment	NA
Ethics oversight	NA

Note that full information on the approval of the study protocol must also be provided in the manuscript.

Field-specific reporting

Please select the one below that is the best fit for your research. If you are not sure, read the appropriate sections before making your selection.

☐ Life sciences ☐ Behavioural & social sciences ☒ Ecological, evolutionary & environmental sciences

For a reference copy of the document with all sections, see nature.com/documents/nr-reporting-summary-flat.pdf

Ecological, evolutionary & environmental sciences study design

All studies must disclose on these points even when the disclosure is negative.

Study description	We analyze species captures obtained from camera trap surveys established across 10 different forested landscapes across Southeast Asia to assess the change in wildlife activity patterns in response to anthropogenic disturbance at the community, guild and species-levels.
Research sample	For all our analyses, the raw data are exclusively time-stamped camera detections of species. We exclude species that are <1 kg due to high inaccuracies in their species-level identifications across all sites. Next, we utilized the remaining 31,138 detections totaling 57 mammalian species (excluding humans), four terrestrial bird species and two reptilian species to assess the change in wildlife activity patterns to varying levels of anthropogenic disturbance. Please refer to Supplementary Table 2 for species-specific information.
Sampling strategy	Our camera trap survey spans across 10 different forested landscapes in Southeast Asia where 18-78 cameras were left for 60-90 days covering an area of 10 to 813 km² at each landscape. This generated >60,000 independent detections and encompassed >100 species which is more than sufficient to address our hypotheses and carry out the relevant analyses. We do note that for our species-level kernel density analysis, we exclude species with <20 independent detection in both intact and disturbed forests. A similar approach is taken for our species-level multinomial modeling approach where we exclude species <15 independent detections within each diel category (Please see Methods on how we define these).
Data collection	We sample wildlife with camera traps at 10 landscapes in Thailand, Peninsular Malaysia, Singapore, Sumatra, and Malaysian Borneo between 2013 to 2019. We deploy 18-78 passive infrared Bushnell and Reconyx cameras for 60–90 days covering 10 to 813 km² at each landscape. We standardise deployment methods across all landscapes by spacing cameras >500 m in larger forests (>50 km²) and 100–500 m apart in smaller forest patches (e.g., Singapore) and attaching them to trees 0.3 m above ground along natural wildlife trails or hiking paths.
Timing and spatial scale	Camera traps were deployed and collected between 2013-05-29 and 2019-09-26 across all our sites . At each site, we deploy 18-78 passive infrared Bushnell and Reconyx cameras for 60–90 days covering 10 to 813 km². We standardise deployment methods across all landscapes by spacing cameras >500 m in larger forests (>50 km²) and 100–500 m apart in smaller forest patches (e.g., Singapore). Please refer to Supplementary table 1 for site-specific camera deployment information.
Data exclusions	Prior to conducting any analysis, we excluded all species with <1 kg due to high inaccuracies in their species-level identifications. We also excluded domesticated taxon (e.g, cattle, dog, house cats) as this can bias our results. Lastly, we also exclude any captures that has no species-level identifications (except for mousedeers) or any captures that have unclear species identifications.
Reproducibility	Firstly, we note that no experiments has been conducted for the purposes of this study and all data used are obtained from wildlife camera trap monitoring. However, to ensure reproducibility of this study, we have created a general pipeline to guide ecologists and researchers in analyzing activity patterns using camera trap data which can be used for any study site. Here is the link to the code: https://github.com/EcologicalCascadesLab/WildlifeActivityPatterns
Randomization	Randomization is not applicable in our study since the purpose of our study is to examine the change in wildlife activity patterns of the specific communities, guilds, and species selected in resposne to anthropogenic disturbance. Please refer to our Methods for more information.
Blinding	Blinding is not applicable in the context of our study since camera traps are triggered by any movement occurring in front of it regardless of what is moving. All species identifications are assigned by various experts in the field.

Did the study involve field work? ☒ Yes ☐ No

Field work, collection and transport

Field conditions	Our fieldwork were conducted in the tropical rainforests of Southeast Asia which is primarily dominated by by tropical evergreen lowland or hill rainforests with canopy emergent trees dominated by the Dipterocarpaceae family. Annual rainfall for the different survey sites ranges from about 1000 to 3000 mm (refer to Supplementary Table 1 for site specific characteristics).
Location	Our study is comprised of 10 different tropical forest landscapes located in Thailand (Khao Yai National Park & Khao Chong Nature Reserve), Peninsular Malaysia (Pasoh Forest Reserve & Ulu Muda Forest Reserve), Singapore, Sumatra (Mount Leuser National Park, Kerinci Seblat National Park & Bukit Barisan Selatan National Park) and Malaysian Borneo (Lambir Hills National Park & Danum Valley Conservation Area). These camera sites have elevations ranging from 0 m to 1,200 m above sea level. Please refer to Figure 2 for the location of study sites.

Access & import/export	In all our sites, MSL obtained all the necessary permits required for fieldwork by the relevant authorities in their respective countries. The data used for this study is open access and can be accessed via Figshare: https://doi.org/10.6084/m9.figshare.23513412.
Disturbance	Camera-trapping is a non-invasive method of collecting live detection of animals, therefore disturbance caused by our sampling is minimal.

Reporting for specific materials, systems and methods

We require information from authors about some types of materials, experimental systems and methods used in many studies. Here, indicate whether each material, system or method listed is relevant to your study. If you are not sure if a list item applies to your research, read the appropriate section before selecting a response.

Materials & experimental systems

n/a	Involved in the study
☒	☐ Antibodies
☒	☐ Eukaryotic cell lines
☒	☐ Palaeontology and archaeology
☐	☒ Animals and other organisms
☒	☐ Clinical data
☒	☐ Dual use research of concern
☒	☐ Plants

Methods

n/a	Involved in the study
☒	☐ ChIP-seq
☒	☐ Flow cytometry
☒	☐ MRI-based neuroimaging

Animals and other research organisms

Policy information about studies involving animals; ARRIVE guidelines recommended for reporting animal research, and Sex and Gender in Research

Laboratory animals	NA
Wild animals	Included directly in manuscript
Reporting on sex	NA
Field-collected samples	Included directly in manuscript
Ethics oversight	NA

Note that full information on the approval of the study protocol must also be provided in the manuscript.

Plants

Seed stocks	NA
Novel plant genotypes	NA
Authentication	NA
